# Efficacy and safety of antioxidants and dietary therapies for epilepsy: an umbrella meta-analysis

**DOI:** 10.3389/fnut.2025.1723370

**Published:** 2026-01-12

**Authors:** Yingqi Feng, Shanshan Cai, Mengyao Wang, Yujia Guo, Ziyu Zhou, Rutong Wang, Shen Yang

**Affiliations:** 1 Department of Neurology, the First People's Hospital of Xiangtan City, Xiangtan, Hunan, China; 2 Clinical Anatomy and Reproductive Medicine Application Institute, Hengyang Medical School, University of South China, Hengyang, Hunan, China; 3 Division of Biomedical and Life Sciences, Faculty of Health and Medicine, Lancaster University, Lancaster, United Kingdom

**Keywords:** antioxidants, drug-resistant epilepsy, epilepsy, ketogenic diet, umbrella meta-analysis

## Abstract

**Background:**

Epilepsy is a prevalent chronic neurological disorder. A substantial proportion of patients develop drug-resistant epilepsy. Existing antiepileptic drugs are associated with adverse effects and demonstrate limited efficacy in refractory cases. Oxidative stress plays a critical role in the pathogenesis of epilepsy. Antioxidants such as vitamin D, vitamin E, and melatonin, as well as dietary therapies like the ketogenic diet, have garnered attention as potential adjunctive treatments. However, existing studies exhibit significant controversy and heterogeneity.

**Objective:**

To evaluate the efficacy and safety of antioxidants and dietary therapies in the treatment of epilepsy, and to provide evidence-based support for clinical decision-making.

**Methods:**

Systematic searches were conducted in PubMed, Web of Science, Embase, and the Cochrane Library. Eighteen eligible meta-analyses were selected for umbrella review based on predefined criteria. Pooled relative risks (RR) with 95% confidence intervals (CI) were calculated using random-effects or fixed-effects models. Heterogeneity was assessed using the I^2^ statistic, and evidence quality was evaluated with the AMSTAR 2 tool.

**Results:**

Dietary therapies—particularly the Low Glycemic Index Treatment (LGIT)—significantly increased the likelihood of achieving ≥50% (RR: 1.95; 95% CI: 1.58–2.33) and ≥90% (RR: 3.33; 95% CI: 1.51–5.14) seizure reduction. However, dietary therapies were ineffective in achieving seizure freedom (RR: 0.68; 95% CI: 0.00–1.36). In subgroup analyses of ≥50% seizure reduction, antioxidants did not demonstrate significant efficacy. Both antioxidants and dietary therapies significantly increased the overall incidence of adverse events (RR: 1.56; 95% CI: 1.19–1.92). Specifically, the ketogenic diet elevated the risks of dyslipidemia (RR: 3.56; 95% CI: 2.00–5.11), weight loss (RR: 4.80; 95% CI: 3.43–6.17), constipation (RR: 3.02; 95% CI: 1.55–4.48), and kidney stones (RR: 5.24; 95% CI: 3.73–6.74). Melatonin was ineffective in reducing seizure frequency (RR: 0.30; 95% CI: 0.00–0.63). Considerable heterogeneity was observed across studies.

**Conclusion:**

Dietary therapies—notably LGIT—demonstrate clear efficacy in reducing seizure frequency but do not facilitate seizure freedom. Current evidence does not support the use of antioxidants as effective adjunctive therapy for epilepsy. These interventions, particularly the ketogenic diet, are associated with increased risks of adverse effects, necessitating careful benefit–risk assessment in clinical application.

**Systematic review registration:**

https://www.crd.york.ac.uk/PROSPERO/view/CRD420251166437, identifier: CRD420251166437.

## 1 Introduction

Epilepsy is a prevalent chronic neurological disorder affecting approximately 65 million individuals worldwide (1, 2). Nearly 30% of these patients develop drug-resistant epilepsy, which severely compromises their quality of life (3). Currently, antiepileptic drugs remain the primary therapeutic approach; however, long-term use may lead to adverse effects such as cognitive impairment and hepatotoxicity, while offering limited efficacy for refractory cases (4, 5). Oxidative stress plays a pivotal role in the pathogenesis of epilepsy. Seizure activity generates excessive free radicals, impairing the self-protective mechanisms of neurons and perpetuating a vicious cycle (6, 7). Consequently, antioxidants such as vitamin D/E and melatonin have garnered attention as potential neuroprotective agents (8, 9). Concurrently, dietary therapies—particularly the ketogenic diet and its variants (e.g., modified Atkins diet and low-glycemic-index treatment)—have emerged as significant non-pharmacological interventions (10). These diets modulate cerebral function by altering energy metabolism, thereby contributing to seizure control. International guidelines already recommend the ketogenic diet for managing refractory epilepsy in pediatric populations (11).

The primary rationale for the combined evaluation of dietary therapies and antioxidant regimens in this comprehensive assessment lies in their shared mechanistic pathway targeting oxidative stress—a common feature in both epileptogenesis and seizure propagation. Although antioxidants function directly as free radical scavengers, robust evidence indicates that dietary therapies, particularly the ketogenic diet, exert significant indirect antioxidant effects. These effects are mediated through multiple mechanisms, including reduced mitochondrial reactive oxygen species (ROS) production via ketone body metabolism, upregulation of the nuclear factor erythroid 2-related factor 2 (Nrf2) pathway to enhance antioxidant enzyme expression, and attenuation of neuroinflammation (9–11). Despite differing modes of intervention—direct supplementation versus metabolic reprogramming—both approaches ultimately aim to correct the underlying redox imbalance in the epileptic brain. This shared mechanistic foundation, combined with their common clinical positioning as adjunctive therapies for drug-resistant epilepsy and overlapping considerations regarding efficacy variability and side effects, provides a strong and coherent basis for their simultaneous evaluation within a unified framework of evidence synthesis. Such an approach enables a unique comparative analysis of how distinct strategies targeting the same pathological pathway translate into clinical outcomes and safety profiles.

Nevertheless, substantial controversies persist in existing research. Although some studies report significant reductions in seizure frequency (12, 13), the potential for complete seizure remission remains uncertain. Clinical outcomes of antioxidant interventions exhibit considerable variability across studies (14, 15). Additionally, the ketogenic diet may induce side effects such as constipation and dyslipidemia (16), necessitating careful risk–benefit evaluation. The existing literature demonstrates heterogeneous quality, leading to inconsistent conclusions. A systematic analysis critically evaluating the efficacy of antioxidants and dietary therapies in epilepsy management is essential to elucidate their potential benefits and resolve discrepancies in current evidence.

## 2 Methods

This study has been prospectively registered for an umbrella meta-analysis on the International Prospective Register of Systematic Reviews (PROSPERO) (Registration No: CRD420251003717). The review strictly adheres to the PRISMA (Preferred Reporting Items for Systematic Reviews and Meta-Analyses) statement and the Cochrane Handbook for Systematic Reviews of Interventions (17, 18).

### 2.1 Inclusion criteria

The included studies were screened according to the following PICOS criteria:

P (Population): Patients with epilepsy.

I (Intervention): Use of antioxidants (such as vitamin D, vitamin E, melatonin) or adoption of dietary therapies (such as ketogenic diet, modified ketogenic diet, low glycemic index therapy).

C (Comparison): Placebo group or regular diet group.

O (Outcome): The primary study outcomes included: ≥50% reduction rate of epileptic seizures, ≥90% reduction rate of epileptic seizures, seizure - free rate, and total incidence of adverse events.

S (Study Design): An umbrella meta-analysis of relevant meta–analyses.

### 2.2 Search strategy and data extraction

A systematic literature search was conducted using PubMed, Web of Science, Embase, and the Cochrane Library databases, with the search period up to September 2, 2025. The search strategy combined Medical Subject Headings (MeSH) and keywords; detailed search strategies are provided in the Supplementary materials. The detailed search strategy is provided in Appendix A.

Two reviewers (RTW and YQF) independently screened titles and abstracts based on predefined criteria. Full texts of potentially eligible studies were retrieved and assessed for final inclusion by the same reviewers. Discrepancies were resolved through consensus or consultation with a third reviewer (SY). Data extracted from included studies comprised publication year, sample size, region, duration of antioxidant or dietary supplementation, odds ratios (ORs), relative risks (RRs), and their corresponding 95% confidence intervals (CIs). To ensure consistency in data analysis, all ORs were uniformly converted to RRs.

The retrieval strategy aims to comprehensively cover relevant content by integrating MeSH terms with free - text keywords related to “epilepsy,” “seizure,” “antioxidants,” and “dietary therapy.” To ensure the reproducibility of the research, Appendix A provides the complete electronic retrieval strategies for all databases, including PubMed, Web of Science, Embase, and the Cochrane Library.

### 2.3 Methodological quality assessment and evidence grading

Two evaluators (RTW and MYW) independently assessed the methodological rigor and quality of the included meta-analyses using A MeaSurement Tool to Assess systematic Reviews (AMSTAR) 2 (19). The AMSTAR 2 tool consists of 16 items, each rated as “yes,” “partial yes,” “no,” or “not applicable.” Additionally, the quality of evidence was categorized into four levels: “very low,” “low,” “moderate,” and “high,” as detailed in Table 1.

**Table 1:** The results of quality assessment included meta-analyses based on a measurement tool to assess systematic reviews 2 questionnaire.

Study	Q1	Q2	Q3	Q4	Q5	Q6	Q7	Q8	Q9	Q10	Q11	Q12	Q13	Q14	Q15	Q16	Overall
Mutarelli, et al. (42)	√	√	√	√	√	√	√	√	√	×	√	√	√	√	√	√	High
Henderson et al. (43)	√	×	×	√	×	×	×	√	×	×	√	×	×	√	×	√	Low
Pizzo et al. (44)	√	×	√	√	√	√	√	√	√	×	√	×	×	√	√	√	High
Liu et al. (45)	×	×	×	√	√	√	×	√	√	×	√	×	×	√	√	√	Low
Sharawat et al. (46)	×	×	×	√	√	×	×	√	√	×	√	×	×	√	√	√	Low
Zhang et al. (47)	×	×	×	√	√	√	×	√	√	×	√	×	×	√	√	√	Low
Sourbron et al. (48)	√	√	√	√	√	√	×	√	√	×	√	×	×	√	×	√	Moderate
Zhu et al. (49)	√	√	√	√	√	√	√	√	√	×	√	×	×	√	√	√	High
Ranganathan et al. (50)	√	×	√	√	√	√	√	√	√	×	√	×	×	√	×	√	High
Manral et al. (51)	√	√	√	√	×	×	×	√	√	×	√	×	×	√	√	√	Moderate
Martin-McGill et al. (52)	√	√	√	√	√	√	√	√	√	√	√	×	×	√	×	√	High
Abbasi et al. (53)	√	√	√	√	√	√	√	√	×	×	√	×	×	√	×	√	Moderate
Mustafa et al. (54)	√	√	√	√	√	√	×	√	√	√	√	×	×	√	×	√	High
Devi et al. (55)	√	√	√	√	√	√	×	√	√	√	√	×	×	√	×	√	High
Liu et al. (56)	√	×	√	√	√	√	×	√	√	×	√	√	√	√	√	√	High
Meng et al. (57)	√	√	√	√	√	√	√	√	√	√	√	√	√	√	√	√	High
Li et al. (20)	√	√	√	√	√	√	×	√	√	×	√	×	×	√	√	√	High
Liu et al. (58)	√	√	√	√	√	√	×	√	√	×	√	×	×	√	×	√	Moderate

The scoring is based on the AMSTAR 2 tool. Each question (Q1 - Q16) is rated as ‘Yes’ (√), ‘Partially Yes/No’ (Partially √), ‘No’ (X), or ‘Not Applicable’. The overall quality is classified into three levels: high, medium, and low, which are determined by the number of compliant items. The specific definitions of the questions are provided in Section 2.3 of the Methodology section.

It is important to note that the AMSTAR 2 tool is specifically designed to assess whether the included meta - analyses can adequately evaluate the risk of bias in their constituent primary studies (Item 9). Therefore, our quality assessment of meta - analyses actually indirectly encompasses an evaluation of the rigor with which the risk of bias in primary studies is addressed.

### 2.4 Statistical analysis

Statistical analyses were performed using STATA 16.0. A fixed-effects model was applied when the I^2^ statistic was below 50%, while a random-effects model was used when I^2^ exceeded 50% to account for between-study heterogeneity. This model assumes that variations in effect sizes arise from differences in study populations, interventions, and outcome measures. Heterogeneity was quantified using the I^2^ statistic and evaluated in accordance with the Cochrane Handbook for Systematic Reviews of Interventions, the I^2^ statistic, and the Q-test. Elevated heterogeneity was defined as a *p*-value < 0.10 coupled with an I^2^ value > 50%. Subgroup analysis or sensitivity analysis was employed to assess the robustness and stability of the umbrella meta-analysis results.

For the calculation of the confidence interval of RR, we employ either a random - effects model or a fixed - effects model. When the lower limit of the confidence interval turns negative (due to small sample sizes or estimation variability), we truncate it to 0.00 to ensure conceptual correctness. This adjustment does not affect the point estimate or statistical conclusions.

## 3 Results

### 3.1 Study screening and characteristics

**Figure 1:**
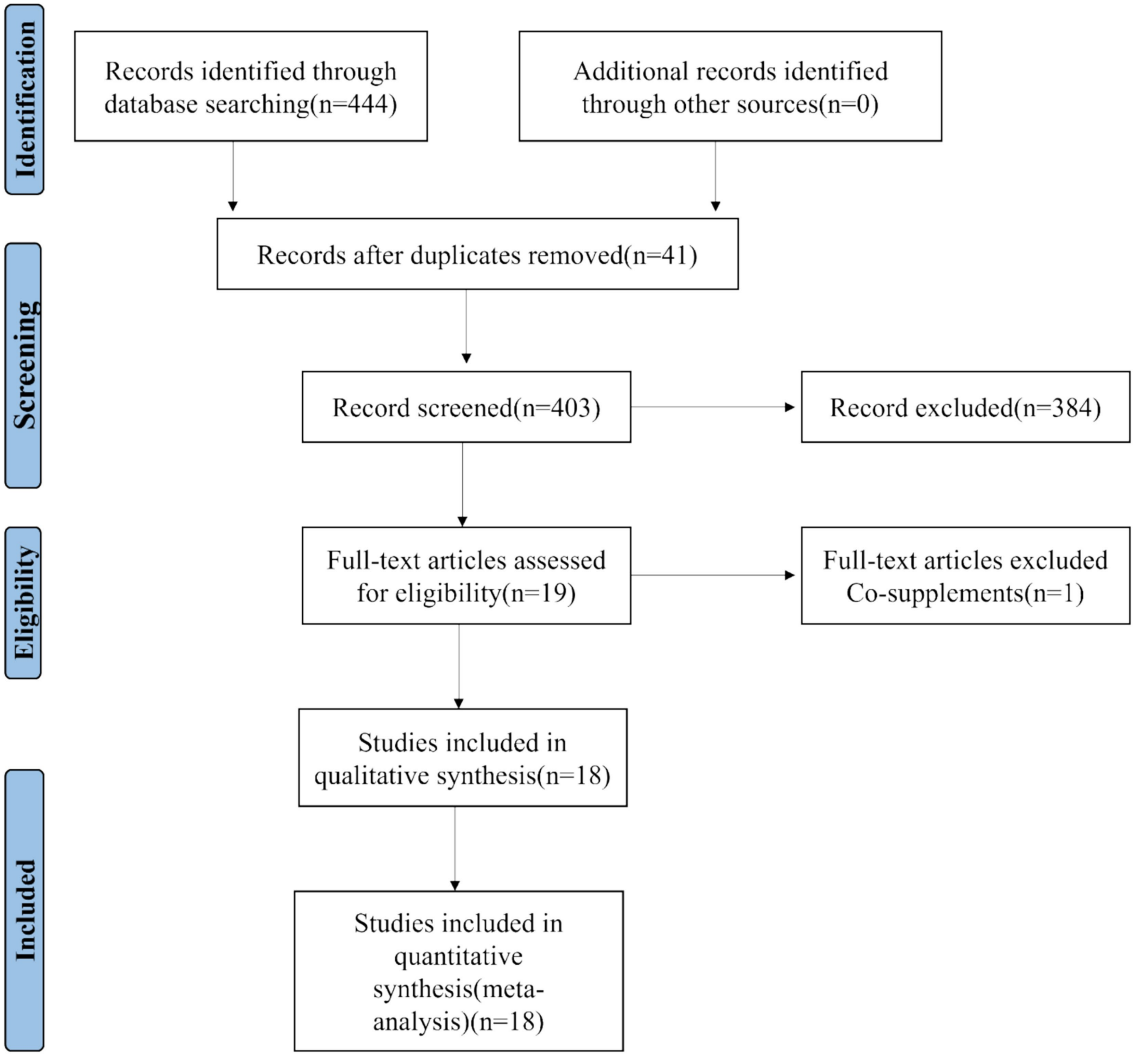
PRISMA flow diagram for study selection in the umbrella meta-analysis on antioxidants and dietary therapies for epilepsy.

A total of 444 studies were retrieved from the initial database search. After removing duplicates and screening titles/abstracts, 19 full - text articles were evaluated. One meta - analysis was excluded due to concerns about the authenticity of its data. Ultimately, 18 studies were included for quantitative synthesis (Figure 1) (19). The details of these studies published between 2005 and 2025 are presented in Table 1.

### 3.2 Results of the umbrella meta - analysis

#### 3.2.1 Efficacy results: seizure reduction and seizure - free status

To comprehensively evaluate the efficacy, we analyzed outcomes reflecting different levels of epilepsy control: seizure reduction ≥50% (indicating clinically significant improvement), seizure reduction ≥90% (approaching seizure - free status, which is a key goal for patients), and seizure - free status (complete cessation of epileptic seizures). These parameters allow for a graded assessment of treatment effects, from significant improvement to ultimate control.

**Figure 2:**
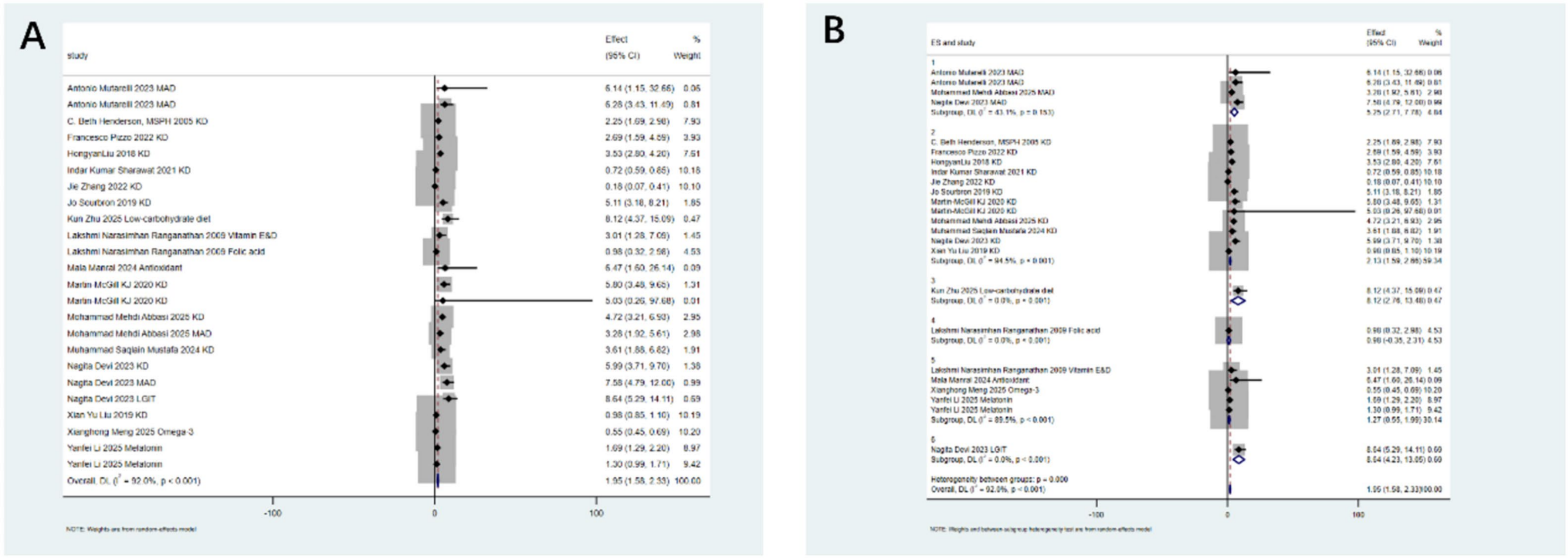
Impact of antioxidants and dietary therapy on seizure reduction. **(A)** Pooled analysis for ≥50% seizure reduction (RR: 1.95; 95% CI: 1.58–2.33); **(B)** subgroup analysis by intervention type, showing LGIT efficacy (RR: 8.64; 95% CI: 4.23–13.05).

Seizure reduction ≥50%: After analyzing 24 studies from 17 articles, the intervention significantly increased the likelihood of achieving a seizure reduction of ≥50% (relative risk RR: 1.95; 95% confidence interval CI: 1.58–2.33). Significant heterogeneity was observed (I^2^ = 92.0%, *p* < 0.001; Figure 2A). Sub - group analysis identified the type of intervention as a key source of heterogeneity (*p* < 0.05). Notably, the low - glycemic index therapy (LGIT) (RR: 8.64; 95% CI: 4.23–13.05) showed excellent efficacy, while the use of antioxidants alone did not show significant benefits (Figure 2B).

**Figure 3:**
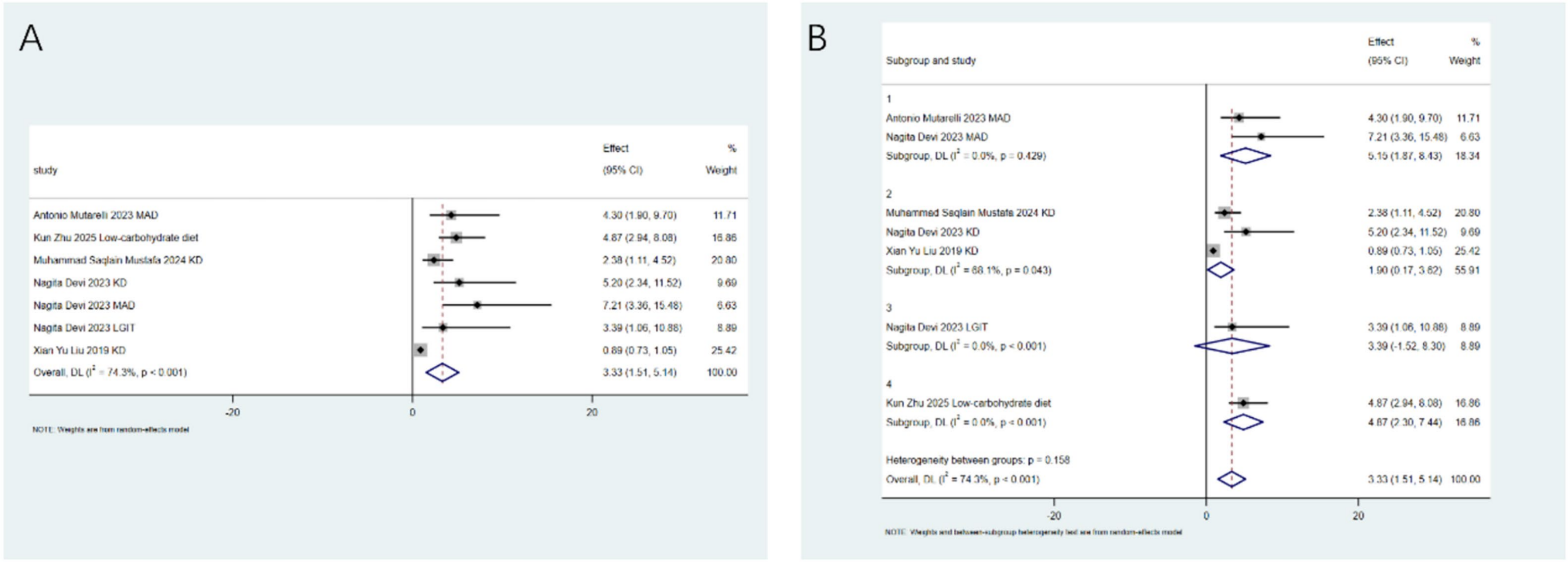
Antioxidant and dietary therapy in achieving ≥90% seizure reduction. **(A)** Pooled analysis (RR: 3.33; 95% CI: 1.51–5.14); **(B)** subgroup analysis highlighting ketogenic diet efficacy (RR: 5.15; 95% CI: 1.87–8.43).

Seizure reduction ≥90%: Focusing on a higher response threshold, after analyzing 7 studies from 5 articles, dietary therapy significantly increased the likelihood of a seizure reduction of ≥90% (RR: 3.33; 95% CI: 1.51–5.14), with moderate heterogeneity (I^2^ = 74.3%, *p* < 0.001; Figure 3A). Sub - group analysis indicated that the ketogenic diet (RR: 5.15; 95% CI: 1.87–8.43) was particularly effective for this outcome (Figure 3B).

**Figure 4:**
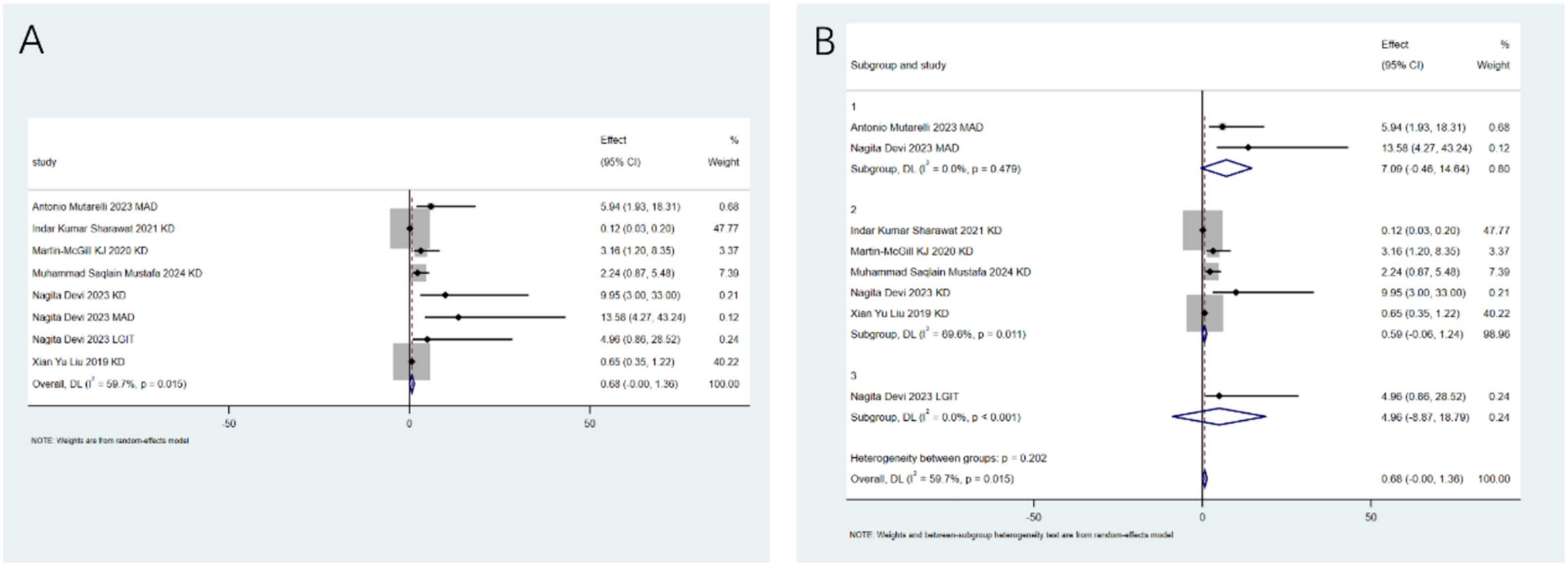
Antioxidants and dietary therapy on seizure freedom. **(A)** Pooled analysis showing no significant effect (RR: 0.68; 95% CI: 0.00–1.36); **(B)** subgroup analysis indicating no intervention type was effective.

Seizure-free status: Contrary to the positive results for seizure reduction, after analyzing 8 studies from 6 articles, dietary therapy was not effective in achieving the ultimate goal of seizure - free status (RR: 0.68; 95% CI: 0.00–1.36), with low heterogeneity (I^2^ = 59.7%, *p* = 0.015; Figure 4A). Sub - group analysis did not identify a specific type of intervention effective for this outcome (Figure 4B). This highlights the crucial difference between significantly reducing seizures and completely stopping them.

#### 3.2.2 Safety results: overall adverse events and specific adverse events

Given the potential risks associated with dietary and antioxidant interventions, we rigorously evaluated safety, examining both the overall burden of adverse events and specific side effects.

**Figure 5:**
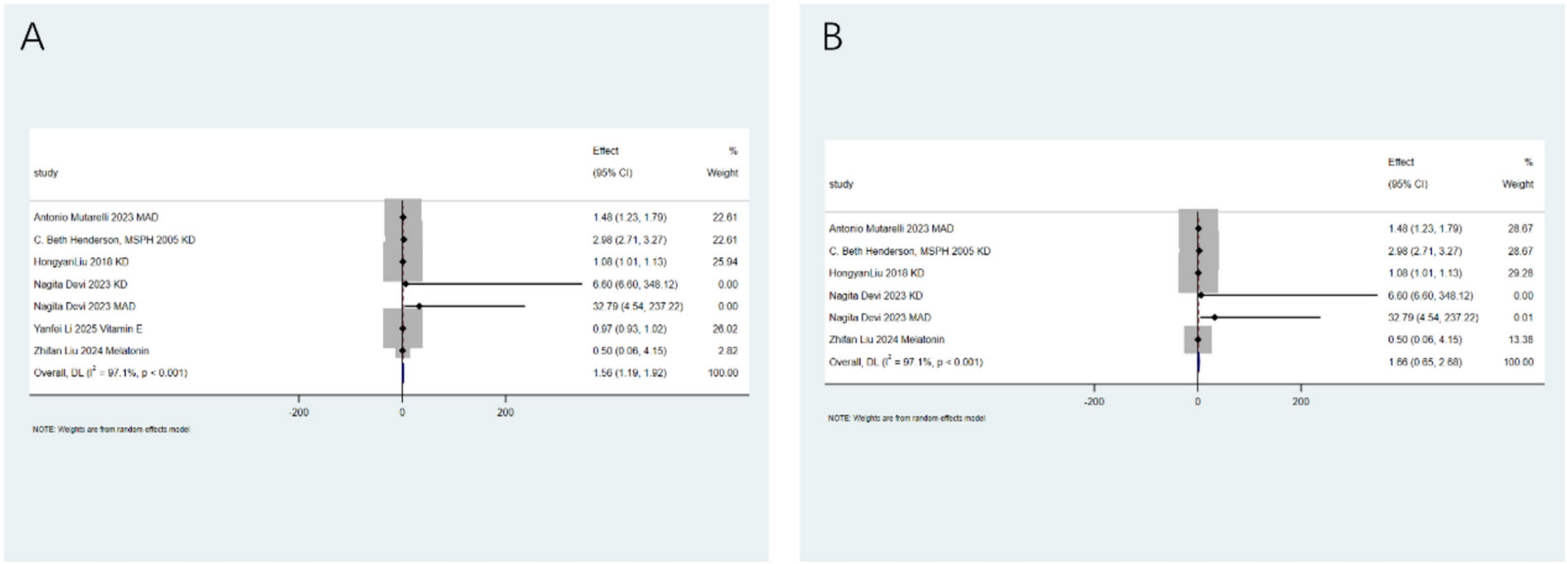
Overall incidence of adverse events. **(A)** Pooled analysis (RR: 1.56; 95% CI: 1.19–1.92); **(B)** sensitivity analysis after excluding outlier study.

Overall incidence of adverse events: A pooled analysis of 7 studies from 6 articles indicated that the intervention significantly increased the overall risk of adverse events (RR: 1.56; 95% CI: 1.19–1.92), with high heterogeneity (I^2^ = 97.1%, *p* < 0.001; Figure 5A). Sensitivity analysis identified one study (20) as a key source of heterogeneity, but excluding this study did not substantially change the results or heterogeneity (I^2^ = 97.1%, *p* < 0.001; Figure 5B). A sensitivity analysis was conducted to assess the robustness of the pooled results of the overall incidence of adverse events, which showed a high degree of heterogeneity (I^2^ = 97.1%). This analysis identified the study by Li et al. (20) as the primary source of the observed heterogeneity. The rationale for exploring its influence lies in the uniqueness of the characteristics of the study population or intervention measures in (20) compared with other studies. However, since excluding (20) did not change the conclusion (the risk estimates remained statistically significant), and the heterogeneity still persisted, and considering that there were no clear methodological flaws in (20), it was decided to retain this study in the main analysis to ensure the comprehensiveness of the evidence. The results of the main analysis and the sensitivity analysis were reported to comprehensively present the stability of the study findings.

**Figure 6:**
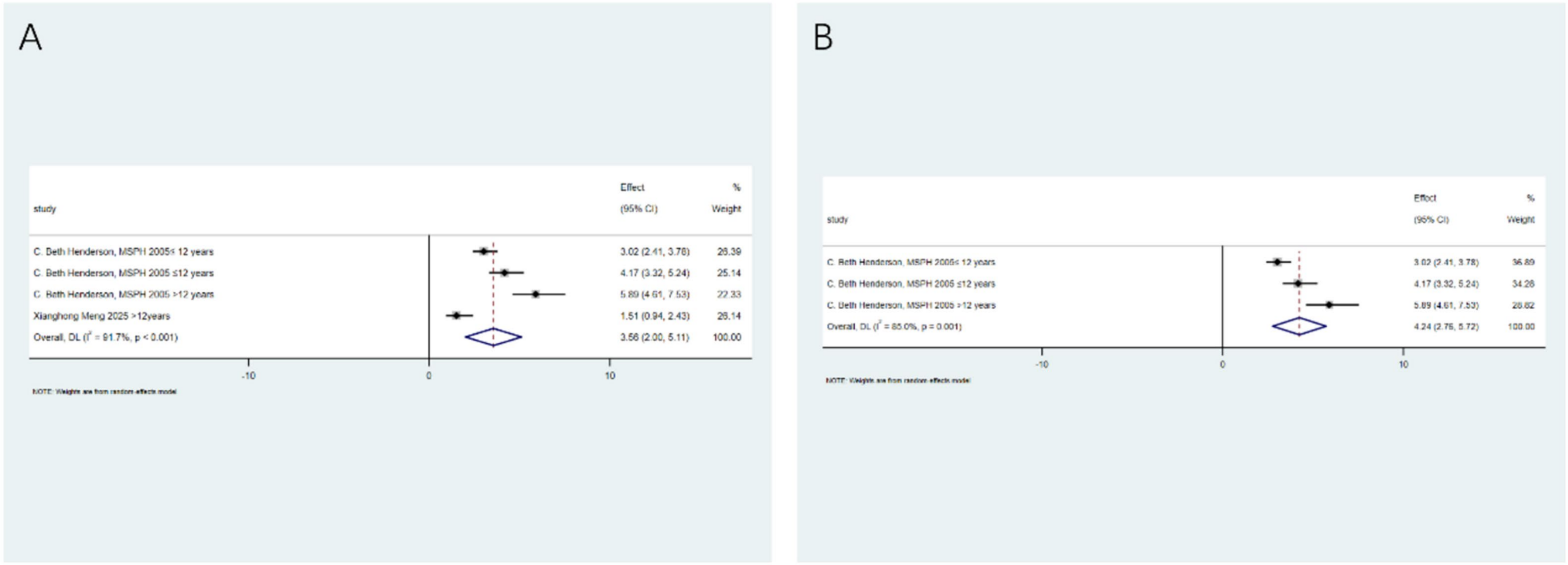
Dyslipidemia risk associated with ketogenic diet. **(A)** Pooled analysis (RR: 3.56; 95% CI: 2.00–5.11); **(B)** Sensitivity analysis confirming robustness.

**Figure 7:**
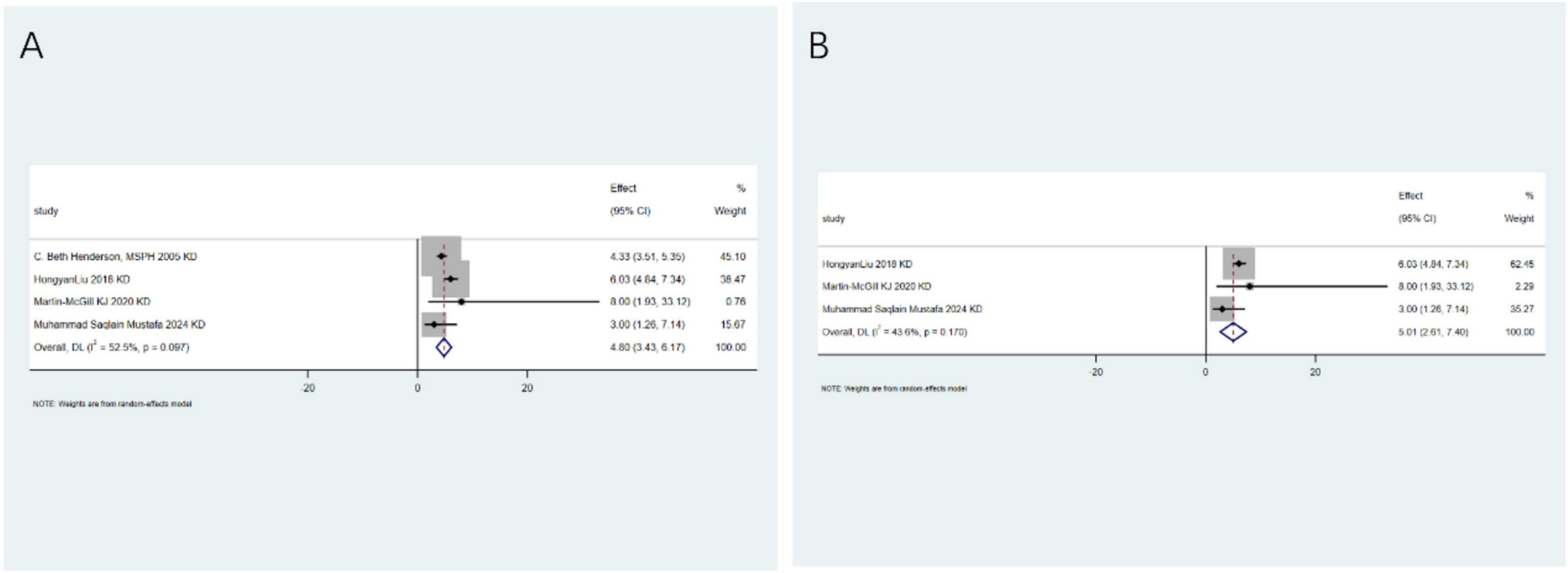
Weight loss as an adverse event of ketogenic diet. **(A)** Pooled analysis (RR: 4.80; 95% CI: 3.43–6.17); **(B)** sensitivity analysis showing consistent effect.

**Figure 8:**
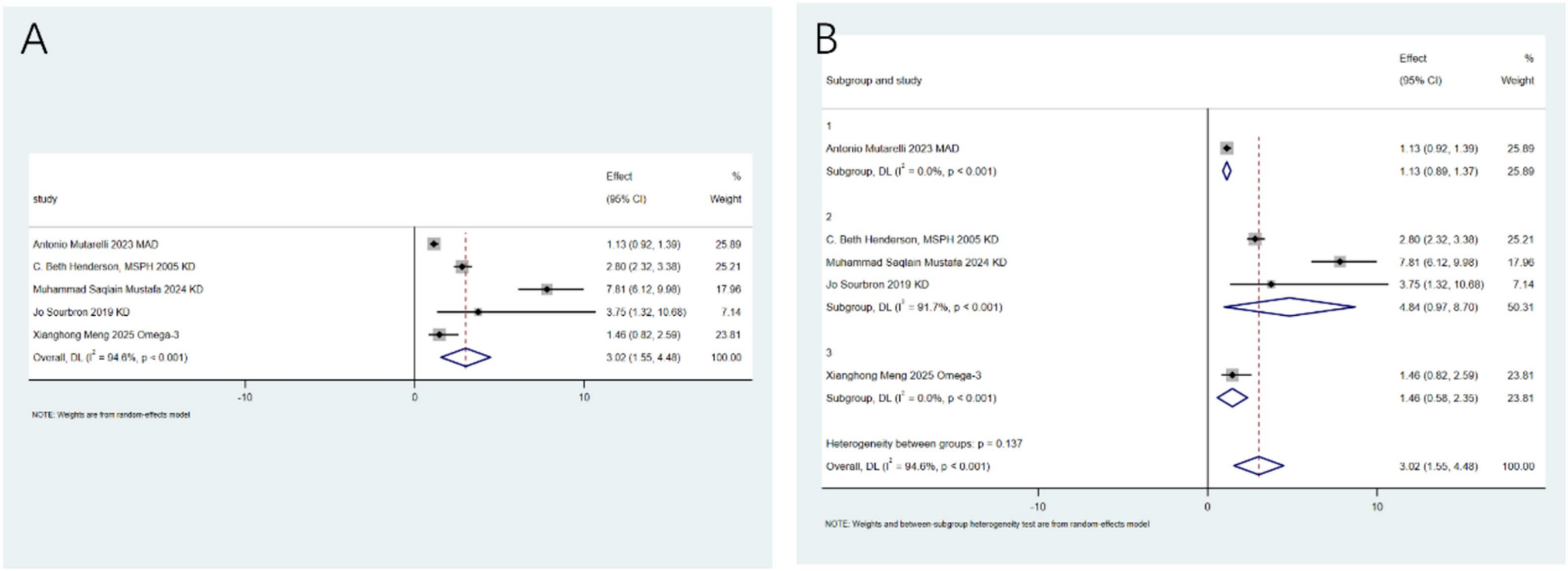
Constipation risk with ketogenic diet. **(A)** Pooled analysis (RR: 3.02; 95% CI: 1.55–4.48); **(B)** Subgroup analysis by diet type.

**Figure 9:**
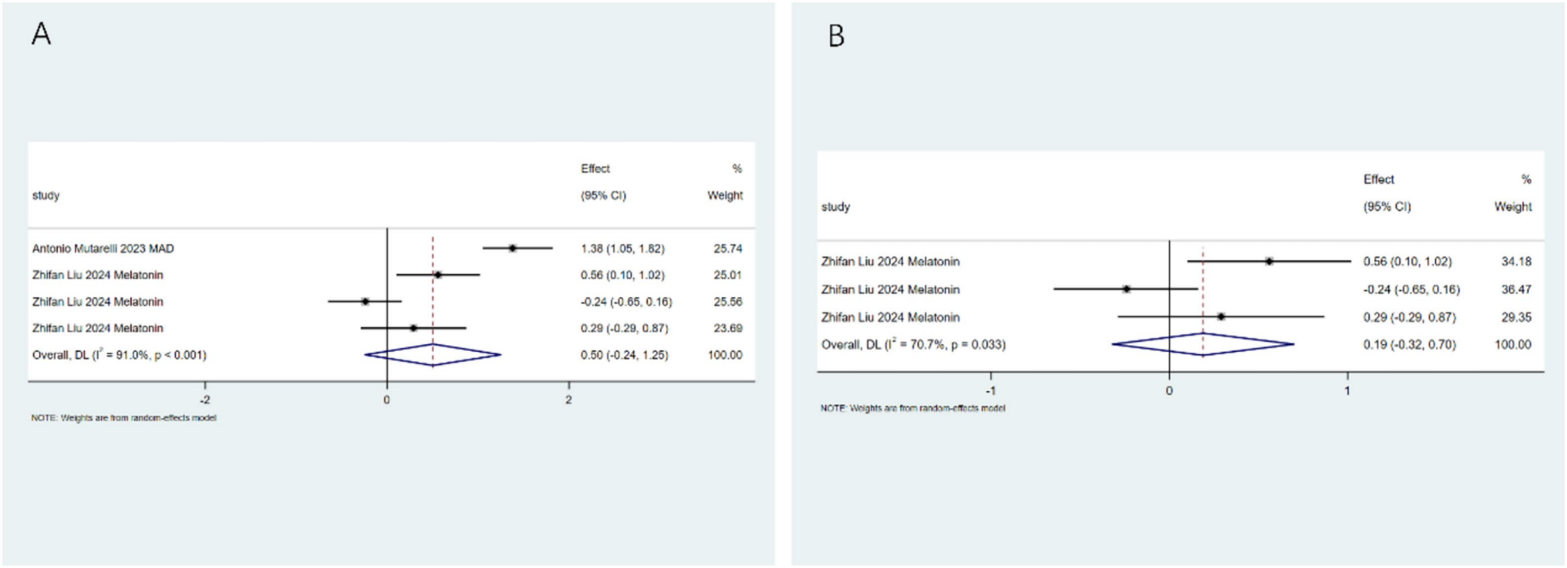
Somnolence risk assessment. **(A)** Pooled analysis showing no significant effect (RR: 0.50; 95% CI: 0–1.25); **(B)** sensitivity analysis confirming result.

**Figure 10:**
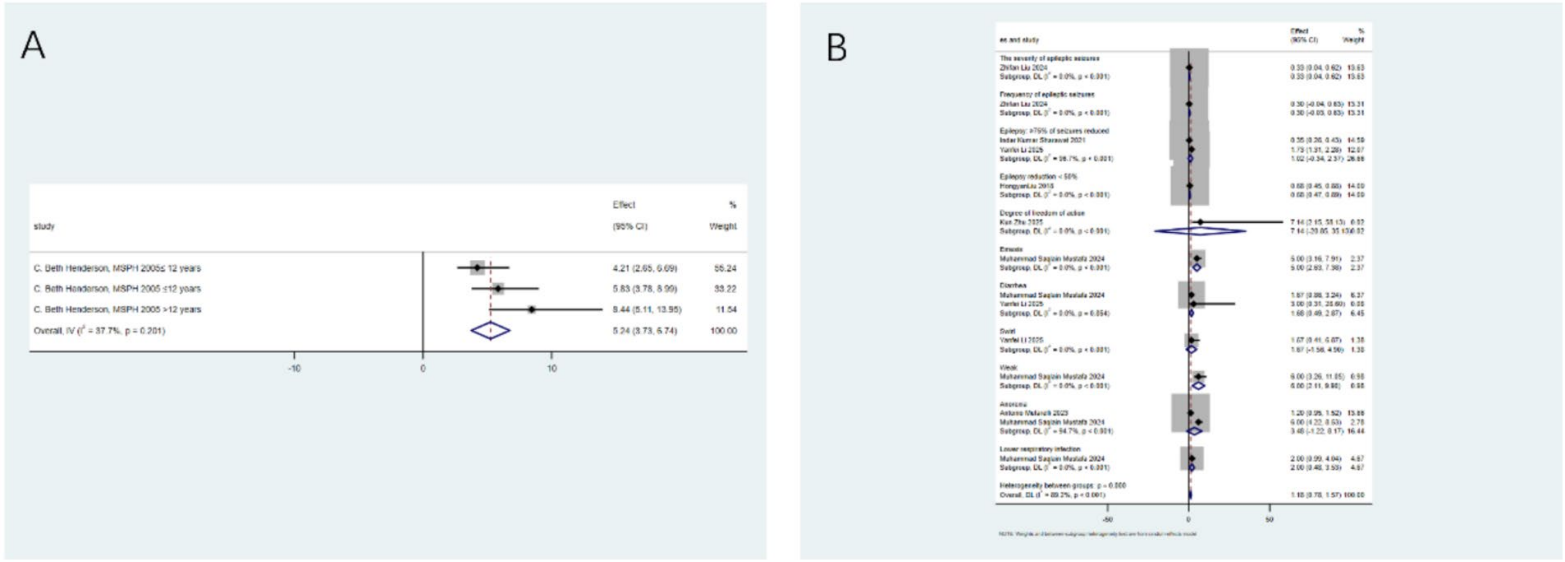
Adverse events of ketogenic diet. **(A)** Kidney stones risk (RR: 5.24; 95% CI: 3.73–6.74); **(B)** Other events including vomiting and fatigue.

Specific adverse events: To clarify the specific risk characteristics, we analyzed individual side-effect profiles. The ketogenic diet is associated with an increased risk of dyslipidemia (RR: 3.56; 95%CI: 2.00–5.11; Figure 6A). After conducting heterogeneity analysis, it was found that the ketogenic diet can increase the risk of dyslipidemia (RR: 4.24; 95%CI: 2.75–5.72; Figure 6B). The ketogenic diet can lead to weight loss (RR: 4.80; 95%CI: 3.43–6.17; Figure 7A). After conducting heterogeneity analysis, it was found that the ketogenic diet can result in weight reduction (RR: 5.01; 95%CI: 2.61–7.40; Figure 7B). The ketogenic diet can cause constipation (RR: 3.02; 95%CI: 1.55–4.48; Figure 8A). After conducting subgroup analysis, it was found that the increase in constipation risk associated with different dietary therapies requires further discussion (Figure 8B). In contrast, this intervention did not significantly increase the risk of somnolence (RR: 0.50; 95%CI: 0.00–1.25; Figure 9A). After conducting heterogeneity analysis, the ketogenic diet may improve somnolence (RR: 0.19; 95%CI: 0.00–0.70; Figure 9B). However, the ketogenic diet is significantly associated with the occurrence of kidney stones (RR: 5.24; 95%CI: 3.73–6.74; Figure 10A).

#### 3.2.3 Other results

For outcomes with limited data (≤2 studies), a descriptive synthesis was performed. The main findings included: Melatonin was ineffective in reducing seizure frequency (RR: 0.30; 95% CI: 0–0.63) but may reduce seizure severity (RR: 0.33; 95% CI: 0.04–0.62). The modified ketogenic diet was effective in reducing seizure frequency (RR: 0.68; 95% CI: 0.45–0.88). The ketogenic diet was associated with vomiting (RR: 5.0; 95% CI: 3.16–7.91) and fatigue (RR: 6.0; 95% CI: 3.26–11.05) but not with diarrhea or anorexia (Figure 10B).

## 4 Discussion

The application of antioxidants and dietary interventions as adjunctive therapies for epilepsy has garnered increasing academic attention (21, 22). While antiepileptic drugs (AEDs) remain the primary treatment modality (23, 24), approximately 30% of patients develop drug-resistant epilepsy, and long-term pharmacotherapy may be associated with a range of adverse effects (25). In recent years, ketogenic diets and their modified variants have been recommended by multiple international guidelines for specific epilepsy syndromes, particularly pediatric refractory epilepsy (22). Additionally, oxidative stress is recognized as playing a significant role in the pathogenesis of epilepsy, leading to growing interest in antioxidant supplementation as a potential adjunctive therapeutic strategy (26). Although studies have explored the roles of antioxidants such as N-acetylcysteine, vitamin E, and coenzyme Q10, as well as various dietary patterns in epilepsy management (27), current randomized controlled trials are limited by small sample sizes, substantial heterogeneity in intervention protocols, and unclear long-term efficacy. Existing meta-analyses also struggle to draw consistent conclusions due to the varying quality of included studies. To address this, the present study employs an umbrella review methodology to comprehensively evaluate 18 meta-analyses published up to September 2025, systematically assessing the efficacy and safety of antioxidants and dietary therapies in epilepsy treatment to provide evidence for clinical practice.

This study reveals that antioxidants and dietary therapies contribute significantly to epilepsy management but may increase the risk of other complications. However, substantial heterogeneity was observed among the meta-analyses. This high degree of heterogeneity necessitates cautious interpretation of the results, particularly when significant variability persists after sensitivity or subgroup analyses. Although previous meta-analyses reported notable overall effects, controversies remain. Discrepancies in outcomes may be attributed to variations in treatment dosage and duration, analytical methods, meta-analysis quality, and study population sizes. The quality of the included meta-analyses was assessed using the AMSTAR 2 tool, which comprises 16 items covering various aspects of meta-analyses. Among all included studies, 10 were high-quality meta-analyses, 4 were of moderate quality, and 4 were of low quality. These findings indicate that the results of this study should be interpreted with caution, underscoring the need for further research to establish definitive conclusions.

A key finding of this umbrella review is the observation of high heterogeneity across multiple outcome measures. Although subgroup analysis identified the type of intervention as an important source, this heterogeneity may stem from the combined effects of clinical and methodological factors. Clinically, the included meta - analyses integrated studies involving patients with different types of epilepsy, varying disease severities, and the use of different concomitant medications. Methodologically, heterogeneity may arise from differences in specific diet therapy regimens, doses and bioavailability of different antioxidants, and disparities in outcome assessment criteria among the original studies. Recognizing these multifaceted sources reinforces the interpretation that the overall effect estimates should be regarded as averages across a range of scenarios, which necessitates caution and an individualized approach in clinical application.

The results of this umbrella meta-analysis highlight the complexity of the role of antioxidants and dietary therapies in epilepsy management, with some findings revealing thought-provoking discrepancies from current research trends or expectations. First, the most notable paradox concerns the seizure-free rate—dietary therapies showed no significant effect on this core efficacy outcome (RR: 0.68; 95% CI: 0.00–1.36). This contrasts with reports from multiple high-quality randomized controlled trials, observational studies, and some of the included meta-analyses demonstrating significant seizure-free outcomes induced by ketogenic diets—particularly the classic ketogenic diet and modified Atkins diet—in specific epilepsy syndromes such as Dravet syndrome, Lennox–Gastaut syndrome, and glucose transporter 1 deficiency syndrome (28, 29). This discrepancy may reflect heterogeneity in patient baseline characteristics (e.g., epilepsy type, degree of drug resistance), strictness of dietary protocol adherence, treatment duration, or definitions of “seizure-free” across studies included in this umbrella review (I^2^ = 59.7%). Subgroup analyses failed to identify responsive subgroups, suggesting that this overall null effect may result from offsetting effects across studies rather than a uniform treatment effect across populations. This underscores the importance of precisely identifying epilepsy subpopulations that respond to specific dietary interventions.

Second, conflicting results were observed regarding the efficacy of antioxidants. Although antioxidants and dietary therapies collectively showed a significant effect in reducing seizure frequency by ≥50% (RR: 1.95; 95% CI: 1.58–2.33), subgroup analysis clearly indicated that antioxidants alone did not improve this outcome (Figure 2B). This contrasts with extensive preclinical research supporting the critical role of oxidative stress in epileptogenesis and some clinical trials reporting positive results for vitamin E, melatonin, N-acetylcysteine, and others (21). Furthermore, the conclusion drawn from our subgroup analysis, which indicates that the use of antioxidants alone does not show significant benefits in reducing seizures by ≥50%, should be interpreted with caution, taking into account physiological and pharmacokinetic variability. The lack of efficacy observed at the population level may mask the potential effectiveness in specific sub - populations. Factors such as inter - individual differences in baseline oxidative stress status, genetic polymorphisms affecting drug metabolism (e.g., metabolism of melatonin or vitamins), bioavailability of different antioxidant formulations (e.g., natural vs. synthetic vitamin E), and differences in dosing regimens across major studies can significantly influence treatment responses. The observed high heterogeneity may partly reflect this underlying variability. Therefore, the current evidence does not support the effectiveness of antioxidants as a uniform intervention. However, this does not rule out the possibility that precision - targeted treatment approaches considering patient - specific factors and optimal pharmacokinetic properties may yield therapeutic effects. This highlights a key direction for future research. This contradiction may stem from several key factors: (a) substantial variations in the type, dose, route of administration, and treatment duration of antioxidants in the original studies included in the meta-analyses, leading to diluted effects; (b) potential differences in the dependence on oxidative stress and responsiveness to antioxidants across different epilepsy types; (c) most existing studies having small sample sizes and short follow-up periods, which may be insufficient to detect clinical benefits of antioxidants—particularly neuroprotective effects—especially when combined with potent antiepileptic drugs, where adjunctive effects may be masked. Additionally, individual reports indicated that melatonin did not significantly improve seizure frequency (RR: 0.30; 95% CI: 0–0.63), and antioxidants did not significantly terminate seizures rapidly (RR: 1.02; 95% CI: 0.00–2.37), further supporting the finding of inconsistent clinical benefits from antioxidants in this umbrella review. This contrasts with some preclinical and limited clinical data suggesting beneficial effects (14) and emphasizes the need for in-depth research on specific antioxidant molecules, dosing regimens, and target populations. We recognize that the reporting of adverse events, particularly in the context of the ketogenic diet, relies on studies that include both pediatric and adult populations without age stratification. For instance, outcomes such as dyslipidemia (RR: 3.56) and weight loss (RR: 4.80) are derived from aggregated mixed - data across different age groups, which may limit in - depth understanding of age-specific risks. This lack of stratification precludes the drawing of clear conclusions regarding whether there are differences in adverse reactions between children and adults. Future meta-analyses should prioritize subgroup analysis by age to clarify these aspects.

Molecules such as glutathione and vitamin E protect neurons from oxidative stress damage by scavenging reactive oxygen species (ROS) and inhibiting lipid peroxidation (30–32). Among them, fat-soluble antioxidants like vitamin E primarily maintain cell membrane stability, while water-soluble antioxidants like vitamin C synergistically regenerate oxidized glutathione and enhance the endogenous antioxidant defense system (33). Studies indicate a bidirectional promoting relationship between oxidative stress and seizures: seizure activity exacerbates free radical generation, and free radical accumulation lowers the seizure threshold, creating a vicious cycle (34). In recent years, various dietary intervention strategies have demonstrated antiepileptic potential. The ketogenic diet reduces mitochondrial ROS production by inducing ketone metabolism and upregulates the nuclear factor erythroid 2-related factor 2 (Nrf2) pathway to enhance antioxidant enzyme expression (35, 36). Furthermore, plant-based antioxidants such as flavonoids not only directly neutralize free radicals but also modulate NF-κB and Nrf2 signaling pathways, suppressing neuroinflammation and promoting GABAergic neurotransmission (37).

To comprehensively situate our research findings within the context of the evolving field of nutritional neurology, it is crucial to integrate new evidence from large - scale epidemiological and mechanistic studies. Recent analyses of data from the National Health and Nutrition Examination Survey (NHANES) in the United States strongly indicate that in the adult population of the United States, a higher dietary antioxidant quality score and adherence to an overall antioxidant-rich diet are significantly associated with a reduced incidence and risk of epilepsy (38, 39). This population - level evidence highlighting the value of overall dietary patterns contrasts with the limited effects of single antioxidant supplements observed in our analysis, suggesting that the food matrix and nutrient synergy play a crucial role. Meanwhile, mechanistic studies have further elucidated the efficacy of carbohydrate - restricted diets. Research has shown that the main ketone body, *β* - hydroxybutyrate, can increase the ratio of GABA to glutamate in the brain, thereby directly inhibiting excessive neuronal excitation (40). Ecological studies have linked high - fat, low - carbohydrate dietary environments to a reduction in the incidence and prevalence of idiopathic epilepsy, confirming the therapeutic potential of this metabolic intervention (41). Overall, these studies not only confirm the biological plausibility of our research findings but also shift the clinical and research focus of epilepsy management from single micronutrient supplementation to the therapeutic regulation of overall dietary patterns and metabolic status.

By rigorously implementing an umbrella meta-analysis—the highest level of evidence synthesis—this study systematically evaluated evidence from meta-analyses published up to September 2025 investigating antioxidants and various dietary therapies (including ketogenic diets and their modifications, low-carbohydrate diets, etc.) for adjunctive epilepsy treatment. The core strengths of this study lie in its methodological rigor (strict adherence to PRISMA guidelines, prospective PROSPERO registration, independent quality assessment of included meta-analyses using AMSTAR 2) and elevated evidence hierarchy, providing a macro-level overview of the efficacy and safety of this emerging intervention strategy. This study is particularly innovative: it comprehensively analyzes both pharmacologic antioxidants and non-pharmacologic dietary interventions, transcending the narrow focus of previous research; it not only systematically evaluates core efficacy outcomes (such as significant seizure frequency reduction) but also quantitatively assesses up to 10 common and potential adverse effects (from dyslipidemia and weight changes to constipation and kidney stones) with unprecedented detail, substantially filling gaps in safety evidence integration; in the face of widespread significant heterogeneity, it systematically conducted subgroup analyses (by intervention type) and sensitivity analyses (identifying key studies) to explore sources of heterogeneity, offering critical insights into the complexity of the results. Importantly, this study keenly identified and analyzed key clinical paradoxes—although dietary therapies (particularly the modified low-glycemic index treatment), LGIT demonstrated clear efficacy in reducing seizure frequency (≥50%), they showed no significant benefit for the ultimate patient goal of “seizure freedom,” and antioxidants overall did not exhibit the expected benefits. This finding challenges some expectations and prompts deeper consideration of precise patient identification, optimization of specific intervention protocols (e.g., type, dose, duration), and long-term safety concerns. In summary, as a landmark integrative study in this field, this umbrella meta-analysis not only provides a comprehensive overview of efficacy and risks based on current evidence but also offers a critical foundation for clinical decision-making. The controversies and knowledge gaps revealed by this analysis.

## 5 Conclusion

This umbrella meta - analysis synthesizes the existing evidence up to September 2025, with the aim of comprehensively assessing the efficacy and safety of antioxidants and dietary therapies as adjunctive treatments for epilepsy. The research findings suggest that dietary intervention measures (particularly the low - carbohydrate ketogenic diet therapy) are significantly correlated with a reduction in the frequency of epileptic seizures (with a seizure frequency reduction ranging from ≥50% to ≥90%). Nevertheless, it is important to note that the analysis did not detect a significant association between dietary therapy and achieving seizure - freedom. Although the combined use of antioxidants and dietary therapy is generally associated with a decrease in the frequency of epileptic seizures, subgroup analysis reveals that, based on the current evidence, the sole use of antioxidants is not associated with improved epilepsy treatment outcomes.

In terms of safety, these interventions (especially the ketogenic diet) are associated with an elevated overall risk of adverse events and are specifically linked to adverse events such as dyslipidemia, constipation, kidney stones, and weight loss. Given the heterogeneity among the studies and the low - to - moderate certainty of the underlying evidence, the results of this research should be interpreted as highlighting relevant associations rather than establishing clear causal relationships. This indicates that when applying dietary therapy to control the frequency of epileptic seizures in clinical practice, it is essential to carefully balance its proven efficacy against the adverse reactions. Regarding antioxidants, the current evidence does not support their use as an effective adjunctive strategy for epilepsy management.

This study offers a significant foundation for clinical decision - making and emphasizes the necessity for future high - certainty research to accurately identify treatment responders, optimize individualized intervention plans, and further explore long - term safety.

Q1: Did the research questions and inclusion criteria for the review include the components of Patient, Intervention, Comparison, Outcome; Q2: Did the report of the review contain an explicit statement that the review methods were established prior to the conduct of the review, and did the report justify any significant deviations from the protocol; Q3: Did the review authors explain their selection of the study designs for inclusion in the review; Q4: Did the review authors use a comprehensive literature search strategy; Q5: Did the review authors perform study selection in duplicate; Q6: Did the review authors perform data extraction in duplicate; Q7: Did the review authors provide a list of excluded studies and 30/35 justify the exclusions; Q8: Did the review authors describe the included studies in adequate detail; Q9: Did the review authors use a satisfactory technique for assessing the risk of bias (RoB) in individual studies that were included in the review; Q10: Did the review authors report on the sources of funding for the studies included in the review; Q11: If metaanalysis was performed, did the review authors use appropriate methods for the statistical combination of results; Q12: If a meta-analysis was performed, did the review authors assess the potential impact of RoB in individual studies on the results of the meta-analysis or other evidence synthesis; Q13: Did the review authors account for RoB in individual studies when interpreting/discussing the review results; Q14: Did the review authors provide a satisfactory explanation for and discussion of any heterogeneity observed in the review results; Q15: If they performed quantitative synthesis, did the review authors conduct an adequate investigation of publication bias (small-study bias) and discuss its likely impact on the review results; Q16: Did the review authors report any potential sources of conflict of interest, including any funding they received for conducting the review. Q: Question.

## Data Availability

The original contributions presented in the study are included in the article/Supplementary material, further inquiries can be directed to the corresponding authors.
