# Supplementary material for: Efficacy and safety of antioxidants and dietary therapies for epilepsy: an umbrella meta-analysis
Source: Front Nutr. 2026 Jan 12;12:1723370. doi: 10.3389/fnut.2025.1723370 (PMC12832287; doi:10.3389/fnut.2025.1723370)
Supplement: Supplementary file 1 [file Data_Sheet_1.ZIP › Supplementary files/Appendix A.docx]

**Search term**

**Appendix 1. Pubmed search strategy**

Searched 2025/9/2

#1 ((((((((((Epilepsy[MeSH Terms]) OR (Epilepsy[Title/Abstract])) OR (Epilepsies[Title/Abstract])) OR (Seizure Disorder*[Title/Abstract])) OR (Epilepsy, Cryptogenic[Title/Abstract])) OR (Cryptogenic Epilepsies[Title/Abstract])) OR (Cryptogenic Epilepsy[Title/Abstract])) OR (Epilepsies, Cryptogenic[Title/Abstract])) OR (Aura*[Title/Abstract])) OR (Awakening Epilepsy[Title/Abstract])) OR (Epilepsy, Awakening[Title/Abstract]) **217,486**

#2 ("antioxidants"[MeSH Terms] OR "vitamins"[MeSH Terms] OR "vitamin e"[MeSH Terms] OR "ascorbic acid"[MeSH Terms] OR "tocopherols"[MeSH Terms] OR "selenium"[MeSH Terms] OR "zinc"[MeSH Terms] OR "ubiquinone"[MeSH Terms] OR "acetylcysteine"[MeSH Terms] OR "carnitine"[MeSH Terms] OR "melatonin"[MeSH Terms] OR "glutathione"[MeSH Terms] OR "carotenoids"[MeSH Terms] OR "arginine"[MeSH Terms] OR "resveratrol"[MeSH Terms] OR "vitamin d"[MeSH Terms] OR "ergocalciferols"[MeSH Terms] OR "curcumin"[MeSH Terms]) OR ("Antioxidants"[Title/Abstract] OR "Vitamin"[Title/Abstract] OR "vitamin e"[Title/Abstract] OR "tocopherol*"[Title/Abstract] OR "alpha tocopherol*"[Title/Abstract] OR "tocotrienol"[Title/Abstract] OR "vitamin c"[Title/Abstract] OR "ascorbic acid"[Title/Abstract] OR "ascorb*"[Title/Abstract] OR "selenium"[Title/Abstract] OR "selen*"[Title/Abstract] OR "zinc"[Title/Abstract] OR "zinc*"[Title/Abstract] OR "ubiquinone"[Title/Abstract] OR "ubiquinol"[Title/Abstract] OR "coenzyme q10"[Title/Abstract] OR "CoQ10"[Title/Abstract] OR "Acetylcysteine"[Title/Abstract] OR "Carnitine"[Title/Abstract] OR "carnitene"[Title/Abstract] OR "melatonin"[Title/Abstract] OR "Glutathione"[Title/Abstract] OR "GSH"[Title/Abstract] OR "carotene"[Title/Abstract] OR "betacarotene"[Title/Abstract] OR "Arginine"[Title/Abstract] OR "resveratrol*"[Title/Abstract] OR primrose"[Title/Abstract] "Curcumin"[Title/Abstract] OR "evening OR "evening primrose oil"[Title/Abstract] OR "vitamin d"[Title/Abstract]) **732,299**

#3 (((((((((Diet[MeSH Terms]) OR (Diet[Title/Abstract])) OR (Mediterranean Diet[Title/Abstract])) OR (Ketogenic Diet[MeSH Terms])) OR (Ketogenic Diet[Title/Abstract])) ) OR (Diets, Mediterranean[Title/Abstract])) OR (Mediterranean Diets[Title/Abstract])) OR (Diets, Ketogenic[Title/Abstract])) OR (Ketogenic Diets[Title/Abstract]) **637,580**

#4 #2 OR #3 **1,311,688**

#5 #1 AND #4 **7,406**

#6 ((Meta-Analysis[Publication Type]) OR (Meta-Analysis[MeSH Terms])) OR (Meta-Analysis[Title/Abstract]) **348,857**

#7 #5 AND #6 **77**

**Appendix 2. Web of Science search strategy**

Searched 2025/9/2

#1 Epilepsy (Topic) or Epilepsies (Topic) or Seizure Disorder* (Topic) or Epilepsy, Cryptogenic (Topic) or Cryptogenic Epilepsies (Topic) or Cryptogenic Epilepsy (Topic) or Epilepsies, Cryptogenic (Topic) or Aura* (Topic) or Awakening Epilepsy (Topic) or Epilepsy, Awakening (Title)

#2 antioxidants (Topic) or vitamins (Topic) or vitamin e (Topic) or ascorbic acid (Topic) or tocopherols (Topic) or selenium (Topic) or zinc (Topic) or ubiquinone (Topic) or acetylcysteine (Topic) or carnitine (Topic) or melatonin (Topic) or glutathione (Topic) or carotenoids (Topic) or arginine (Topic) or resveratrol (Topic) or vitamin d (Topic) or ergocalciferols (Topic) or curcumin (Topic) or Diet (Topic) or Mediterranean Diet (Topic) or Ketogenic Diet (Topic) or Diets, Mediterranean (Topic) or Mediterranean Diets (Topic) or Diets, Ketogenic (Topic) or Ketogenic Diets (Topic)

#3 Meta-Analysis (Topic) or Meta-Analysis (Title)

#4 #1 AND #2 AND #3 **163**

**Appendix 3. Embase search strategy**

Searched 2025/9/2

#1 epilepsy:ab,ti OR epilepsies:ab,ti OR 'seizure disorder*':ab,ti OR 'epilepsy, cryptogenic':ab,ti OR 'cryptogenic epilepsies':ab,ti OR 'cryptogenic epilepsy':ab,ti OR 'epilepsies, cryptogenic':ab,ti OR aura*:ab,ti OR 'awakening epilepsy':ab,ti OR 'epilepsy, awakening':ab,ti **248,815**

#2 antioxidants:ab,ti OR vitamins:ab,ti OR 'vitamin e':ab,ti OR 'ascorbic acid':ab,ti OR tocopherols:ab,ti OR selenium:ab,ti OR zinc:ab,ti OR ubiquinone:ab,ti OR acetylcysteine:ab,ti OR carnitine:ab,ti OR melatonin:ab,ti OR glutathione:ab,ti OR carotenoids:ab,ti OR arginine:ab,ti OR resveratrol:ab,ti OR 'vitamin d':ab,ti OR ergocalciferols:ab,ti OR curcumin:ab,ti OR diet:ab,ti OR 'mediterranean diet':ab,ti OR 'ketogenic diet':ab,ti OR 'diets, mediterranean':ab,ti OR 'mediterranean diets':ab,ti OR 'diets, ketogenic':ab,ti OR 'ketogenic diets':ab,ti **1,519,734**

#3 'meta analysis':ab,ti OR 'meta analysis':it OR 'meta analysis'/exp **469,773**

#4 #1 AND #2 AND #3 **115**

**Appendix 4. Cochranelibrary search strategy**

Searched 2025/9/2

#1 (Alzheimer Disease):ti,ab,kw OR (Alzheimer Syndrome):ti,ab,kw OR (Alzheimer-Type Dementia):ti,ab,kw OR (Alzheimer Type Dementia):ti,ab,kw OR (Dementia, Alzheimer-Type):ti,ab,kw (Word variations have been searched)

#2 (Alzheimer's Diseases):ti,ab,kw OR (Alzheimer Diseases):ti,ab,kw OR (Alzheimers Diseases):ti,ab,kw OR (Alzheimer Dementia):ti,ab,kw OR (Alzheimer Dementias):ti,ab,kw (Word variations have been searched)

#3 (Dementia, Alzheimer):ti,ab,kw OR (Alzheimer's Disease):ti,ab,kw OR (Dementia, Senile):ti,ab,kw OR (Senile Dementia):ti,ab,kw OR (Dementia, Alzheimer Type):ti,ab,kw (Word variations have been searched)

#4 (Alzheimer Type Dementia):ti,ab,kw OR (Sclerosis, Alzheimer):ti,ab,kw OR (Dementia, Primary Senile Degenerative):ti,ab,kw OR (Dementia, Presenile):ti,ab,kw OR (Presenile Dementia):ti,ab,kw (Word variations have been searched)

#5 (Acute Confusional Senile Dementia):ti,ab,kw OR (Senile Dementia, Acute Confusional):ti,ab,kw OR (Alzheimer Disease, Early Onset):ti,ab,kw OR (Early Onset Alzheimer Disease):ti,ab,kw OR (Presenile Alzheimer Dementia):ti,ab,kw (Word variations have been searched)

#6 (Alzheimer Disease, Late Onset):ti,ab,kw OR (Late Onset Alzheimer Disease):ti,ab,kw OR (Alzheimer's Disease, Focal Onset):ti,ab,kw OR (Focal Onset Alzheimer's Disease):ti,ab,kw OR (Familial Alzheimer Disease):ti,ab,kw (Word variations have been searched)

#7 #1 OR #2 OR #3 OR #4 OR #5 OR #6

#8 (Selenium):ti,ab,kw OR (Zinc):ti,ab,kw OR (Ubiquinone):ti,ab,kw OR (Acetylcystein):ti,ab,kw OR (Carnitine):ti,ab,kw (Word variations have been searched)

#9 (Melatonin):ti,ab,kw OR (Glutathione):ti,ab,kw OR (Carotenoids):ti,ab,kw OR (Arginine):ti,ab,kw OR (Resveratrol):ti,ab,kw (Word variations have been searched)

#10 #8 OR #9

#11 #10 AND #7

#12 (Epilepsy):ti,ab,kw (Word variations have been searched)

#13 #7 OR #10 OR #11 OR #12

#14 MeSH descriptor: [Antioxidants] explode all trees

#15 (antioxidants):ti,ab,kw

#16 MeSH descriptor: [Vitamins] explode all trees

#17 (vitamins):ti,ab,kw

#18 MeSH descriptor: [Vitamin E] explode all trees

#19 (vitamin e):ti,ab,kw

#20 (ascorbic acid):ti,ab,kw

#21 MeSH descriptor: [Ascorbic Acid] explode all trees

#22 MeSH descriptor: [Tocopherols] explode all trees

#23 (tocopherols):ti,ab,kw

#24 (selenium):ti,ab,kw

#25 MeSH descriptor: [Selenium] explode all trees

#26 (zinc):ti,ab,kw

#27 MeSH descriptor: [Zinc] explode all trees

#28 (ubiquinone):ti,ab,kw

#29 MeSH descriptor: [Ubiquinone] explode all trees

#30 (acetylcysteine):ti,ab,kw

#31 MeSH descriptor: [Acetylcysteine] explode all trees

#32 (carnitine):ti,ab,kw

#33 MeSH descriptor: [Carnitine] explode all trees

#34 (melatonin):ti,ab,kw

#35 MeSH descriptor: [Melatonin] explode all trees

#36 (glutathione):ti,ab,kw

#37 MeSH descriptor: [Glutathione] explode all trees

#38 (carotenoids):ti,ab,kw

#39 MeSH descriptor: [Carotenoids] explode all trees

#40 MeSH descriptor: [Arginine] explode all trees

#41 (arginine):ti,ab,kw

#42 (resveratrol):ti,ab,kw

#43 MeSH descriptor: [Resveratrol] explode all trees

#44 (vitamin d):ti,ab,kw

#45 MeSH descriptor: [Vitamin D] explode all trees

#46 (ergocalciferols):ti,ab,kw

#47 MeSH descriptor: [Ergocalciferols] explode all trees

#48 (curcumin):ti,ab,kw

#49 MeSH descriptor: [Curcumin] explode all trees

#50 (Diet):ti,ab,kw

#51 MeSH descriptor: [Diet] explode all trees

#52 (Mediterranean Diet):ti,ab,kw

#53 MeSH descriptor: [Diet, Mediterranean] explode all trees

#54 (Ketogenic Diet):ti,ab,kw

#55 MeSH descriptor: [Diet, Ketogenic] explode all trees

#56 #14 OR #15 OR #16 OR #17 OR #18 OR #19 OR #20 OR #21 OR #22 OR #23 OR #24 OR #25 OR #26 OR #27 OR #28 OR #29 OR #30 OR #31 OR #32 OR #33 OR #34 OR #35 OR #36 OR #37 OR #38 OR #39 OR #40 OR #41 OR #42 OR #43 OR #44 OR #45 OR #46 OR #47 OR #48 OR #49 OR #50 OR #51 OR #52 OR #53 OR #54 OR #55

#57 #13 AND #56

#58 (Meta-Analysis):ti,ab,kw

#59 MeSH descriptor: [Meta-Analysis] explode all trees

#60 #58 OR #59

#61 #57 AND #60 **89**
