# Supplementary material for: Efficacy and safety of antioxidants and dietary therapies for epilepsy: an umbrella meta-analysis
Source: Front Nutr. 2026 Jan 12;12:1723370. doi: 10.3389/fnut.2025.1723370 (PMC12832287; doi:10.3389/fnut.2025.1723370)
Supplement: Supplementary file 1 [file Data_Sheet_1.ZIP › Supplementary files/Figure.pptx]

## Slide 1
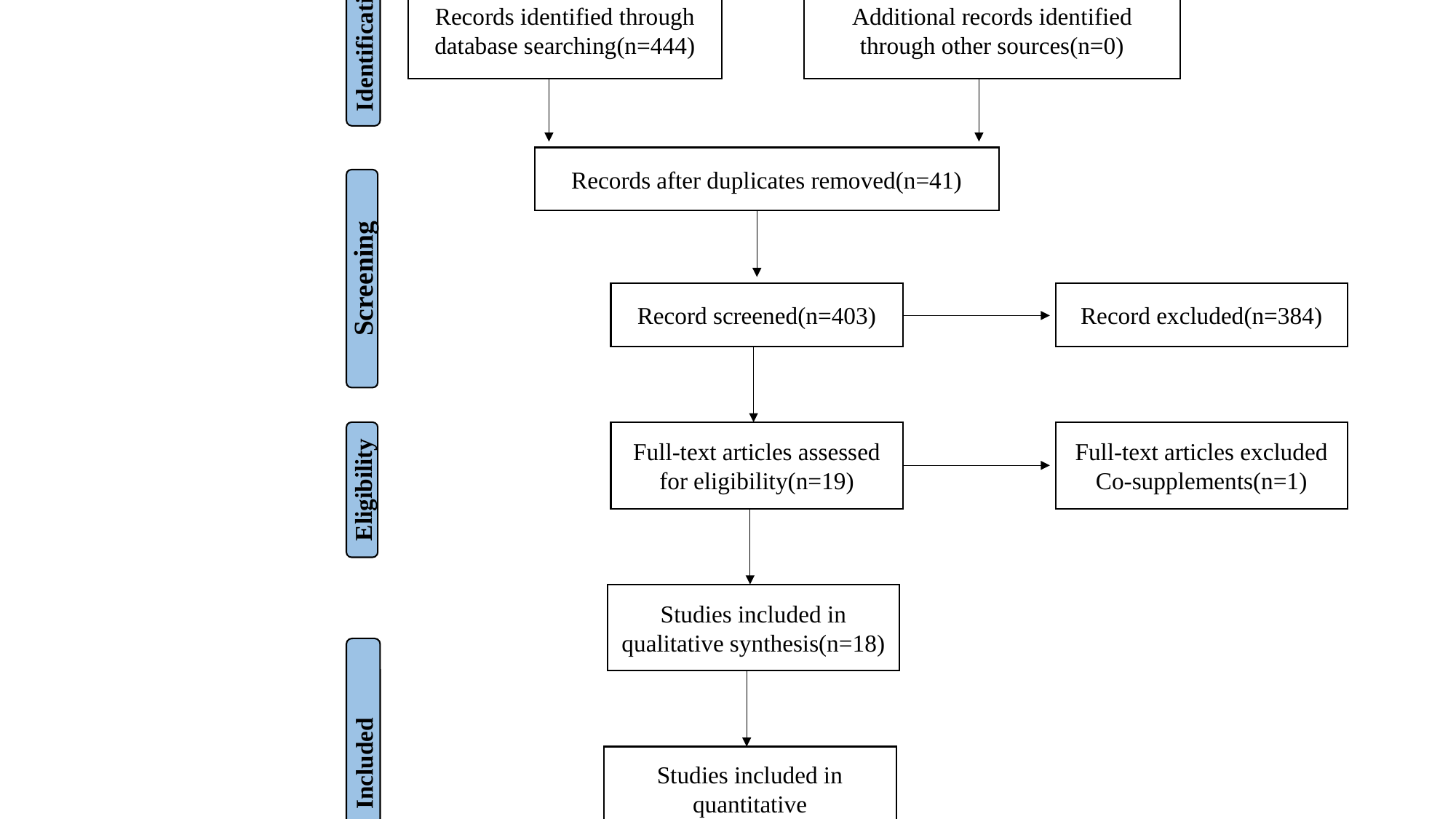

Records identified through database searching(n=444)
Additional records identified through other sources(n=0)
Identification
Records after duplicates removed(n=41)
Screening
Record screened(n=403)
Record excluded(n=384)
Full-text articles assessed for eligibility(n=19)
Full-text articles excluded Co-supplements(n=1)
Eligibility
Studies included in qualitative synthesis(n=18)
Studies included in quantitative synthesis(meta-analysis)(n=18)
Included
Figure 1. Forest plot of the effect of inositol supplementation on gestational diabetes mellitus incidence.

## Slide 2
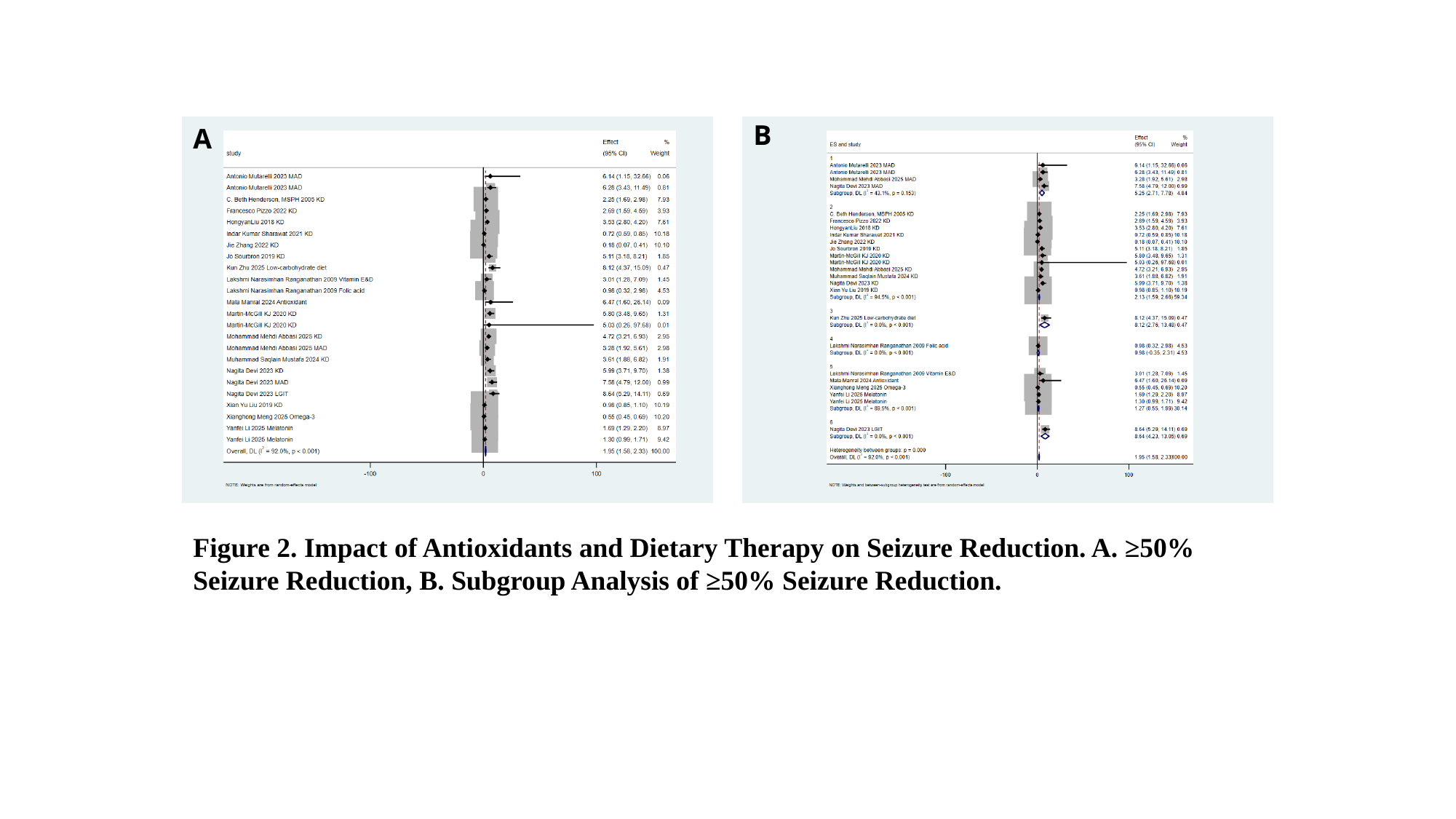

B
A
Figure 2. Impact of Antioxidants and Dietary Therapy on Seizure Reduction. A. ≥50% Seizure Reduction, B. Subgroup Analysis of ≥50% Seizure Reduction.

## Slide 3
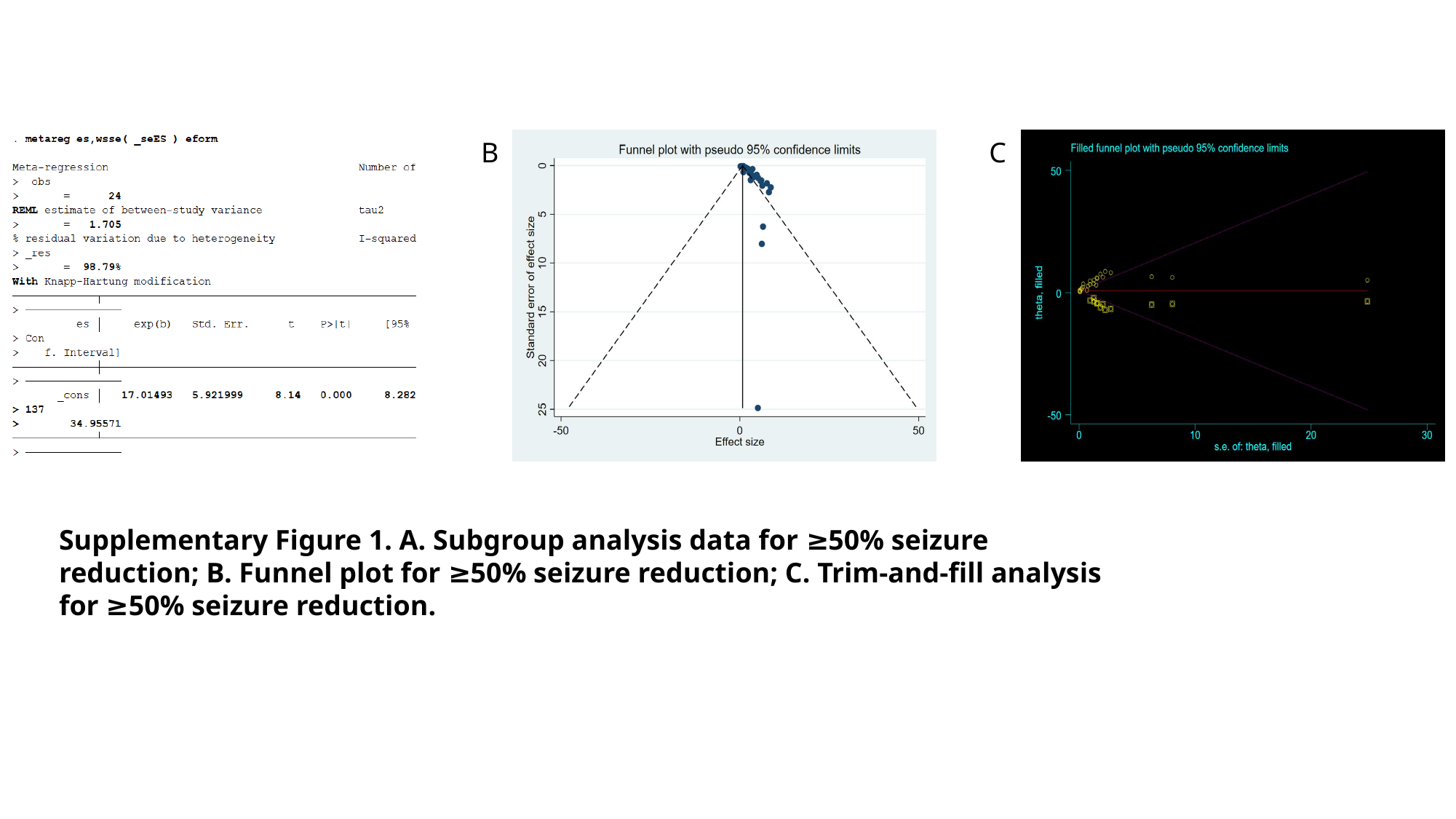

A
B
C
Supplementary Figure 1. A. Subgroup analysis data for ≥50% seizure reduction; B. Funnel plot for ≥50% seizure reduction; C. Trim-and-fill analysis for ≥50% seizure reduction.

## Slide 4
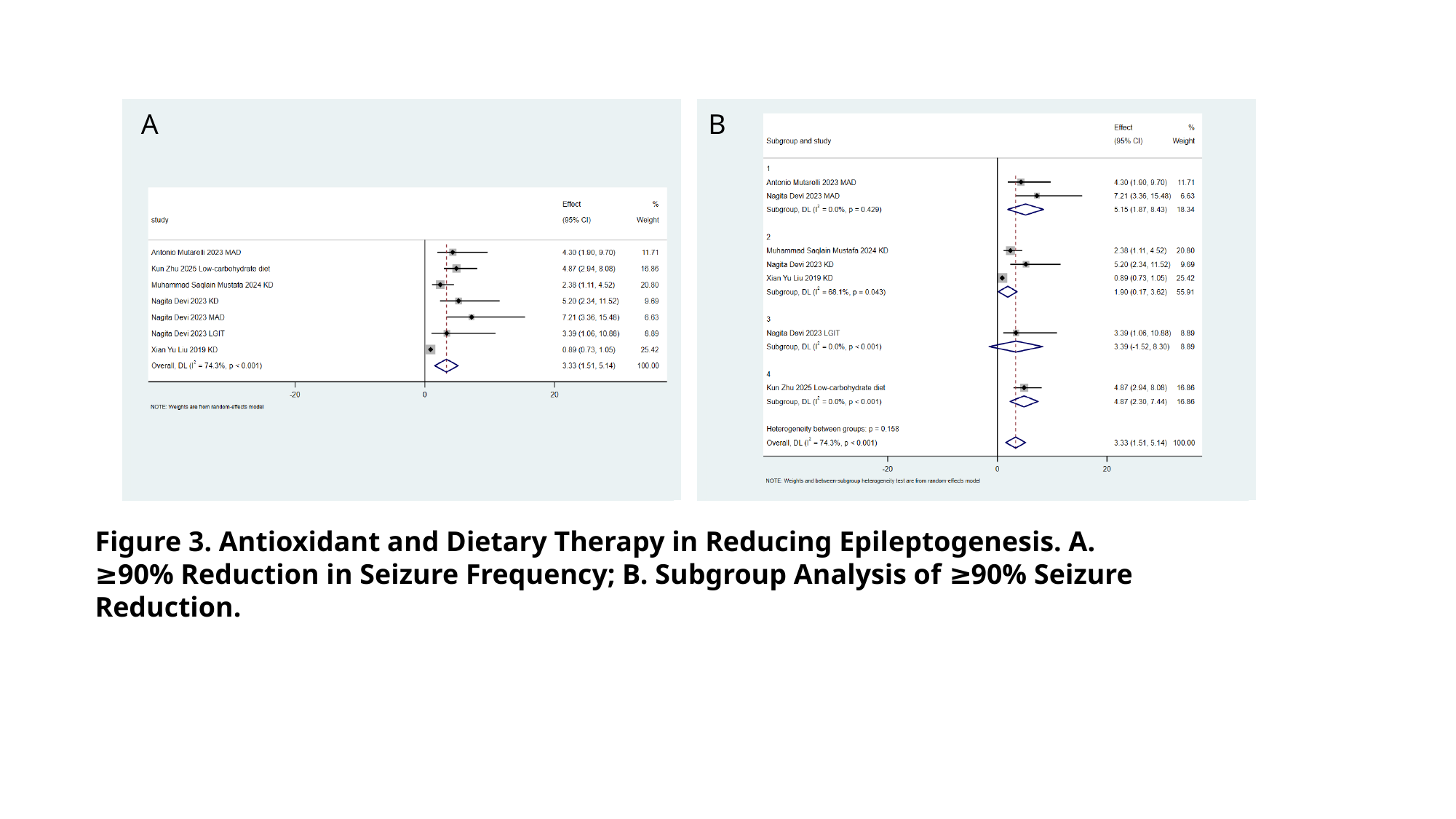

A
B
Figure 3. Antioxidant and Dietary Therapy in Reducing Epileptogenesis. A. ≥90% Reduction in Seizure Frequency; B. Subgroup Analysis of ≥90% Seizure Reduction.

## Slide 5
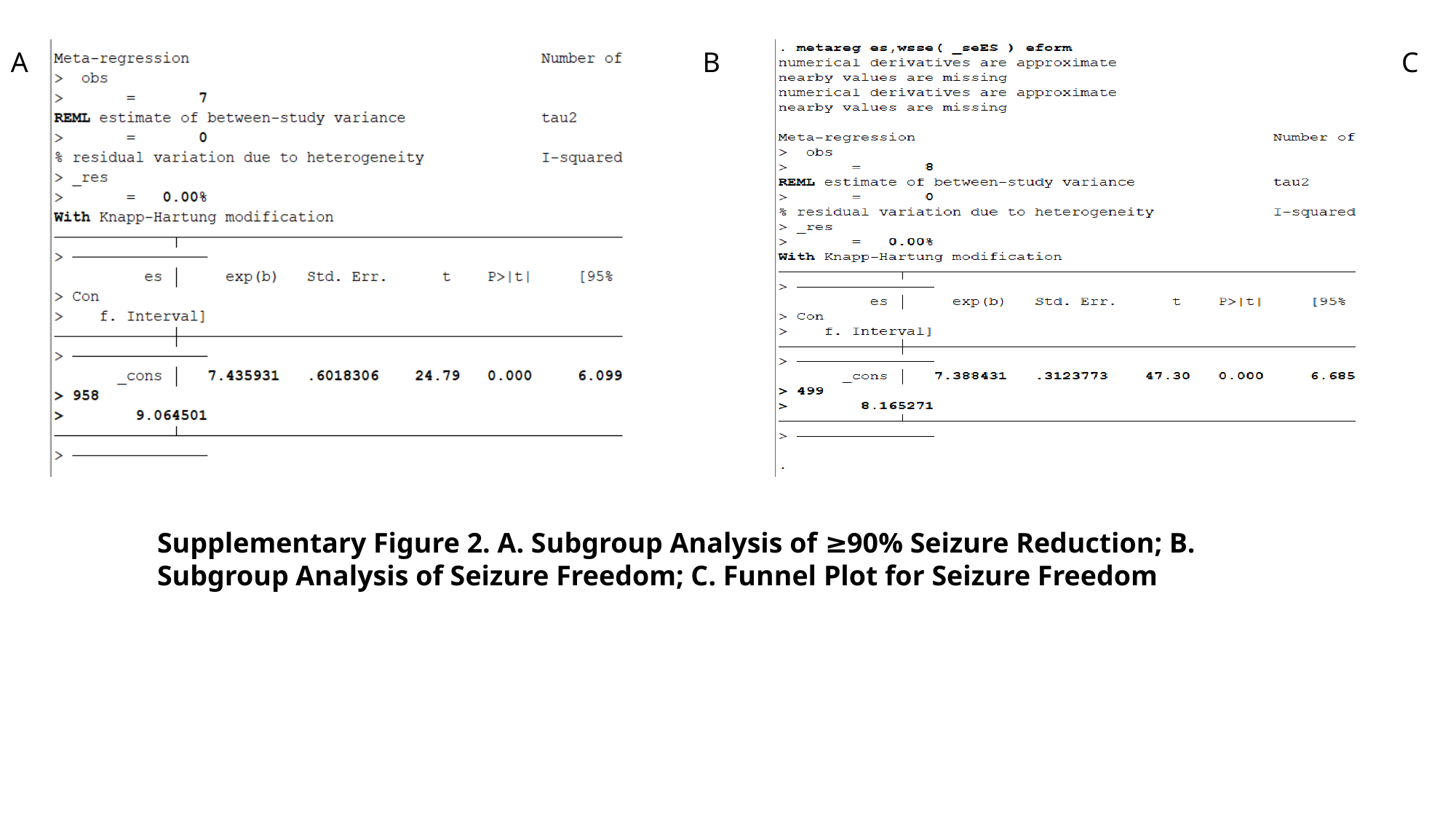

C
A
B
Supplementary Figure 2. A. Subgroup Analysis of ≥90% Seizure Reduction; B. Subgroup Analysis of Seizure Freedom; C. Funnel Plot for Seizure Freedom

## Slide 6
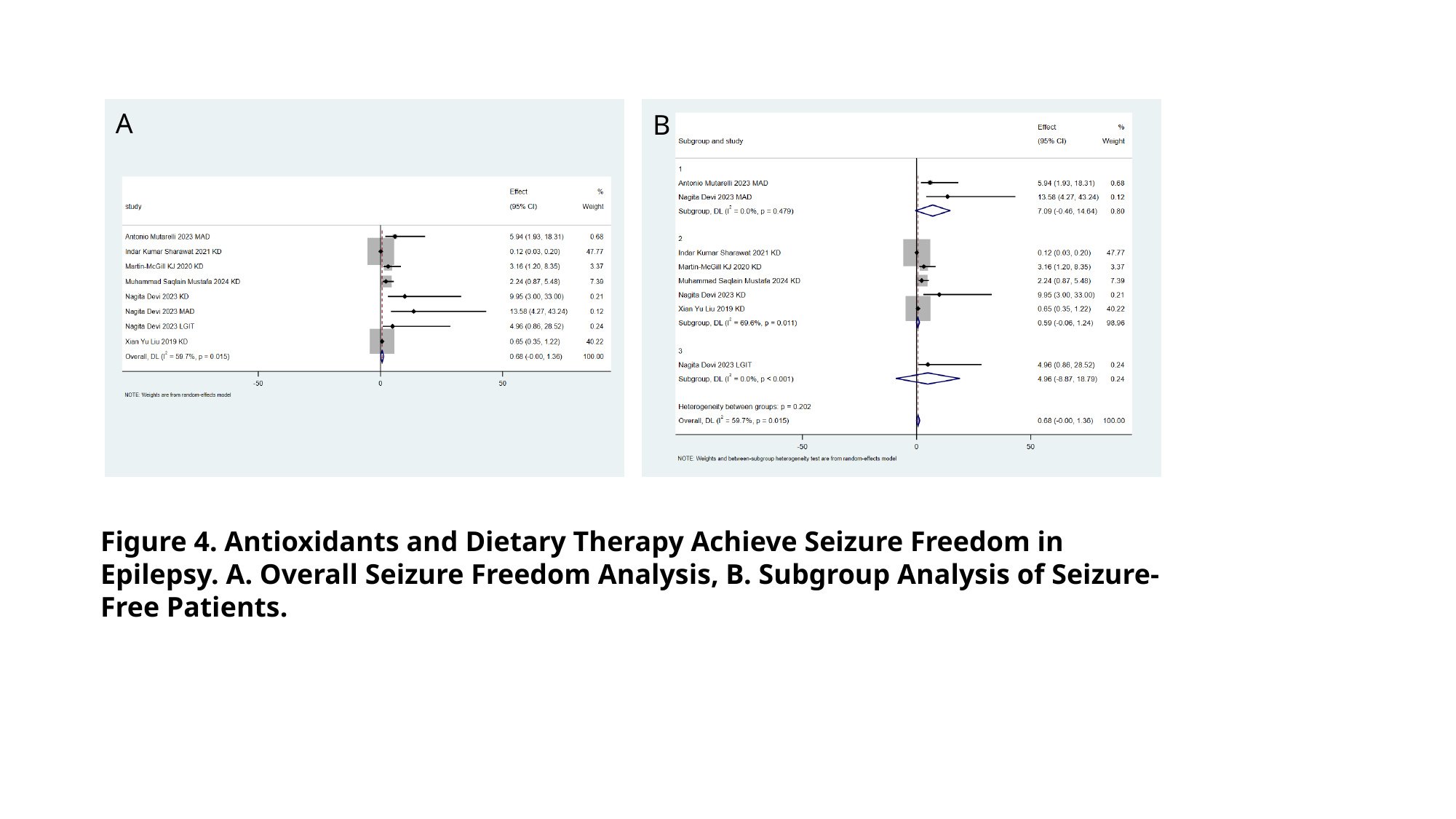

A
B
Figure 4. Antioxidants and Dietary Therapy Achieve Seizure Freedom in Epilepsy. A. Overall Seizure Freedom Analysis, B. Subgroup Analysis of Seizure-Free Patients.

## Slide 7
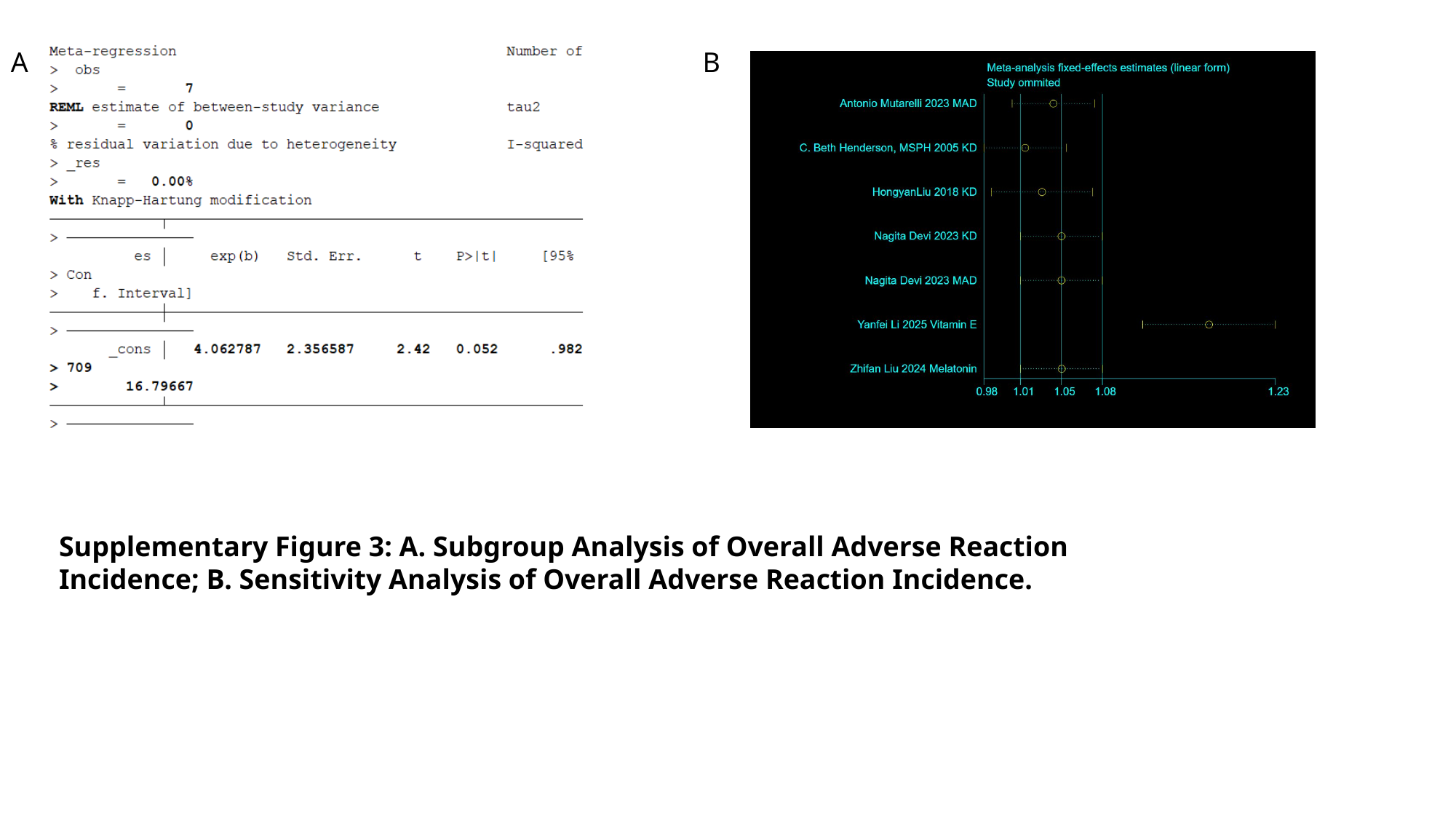

A
B
Supplementary Figure 3: A. Subgroup Analysis of Overall Adverse Reaction Incidence; B. Sensitivity Analysis of Overall Adverse Reaction Incidence.

## Slide 8
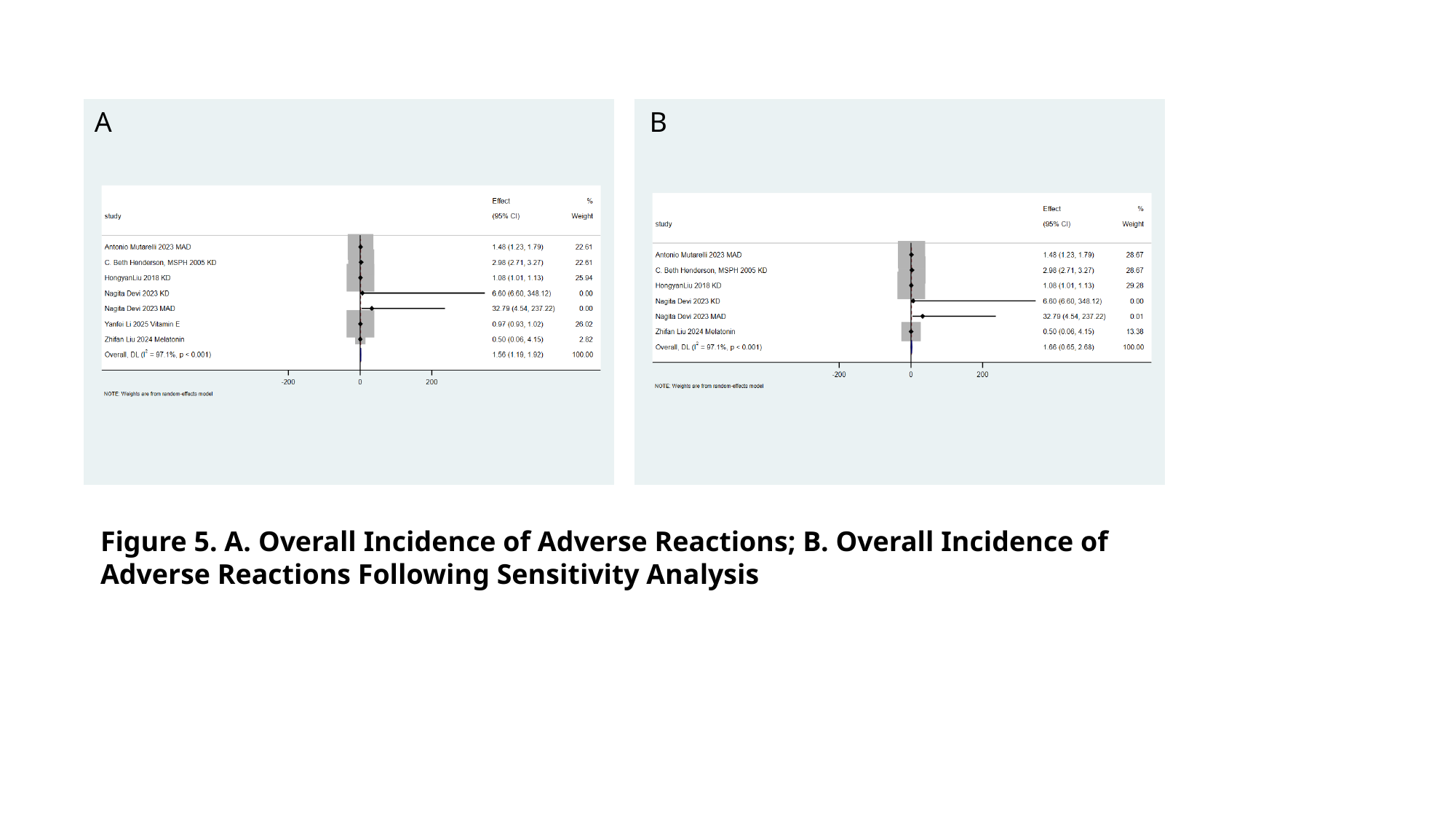

A
B
Figure 5. A. Overall Incidence of Adverse Reactions; B. Overall Incidence of Adverse Reactions Following Sensitivity Analysis

## Slide 9
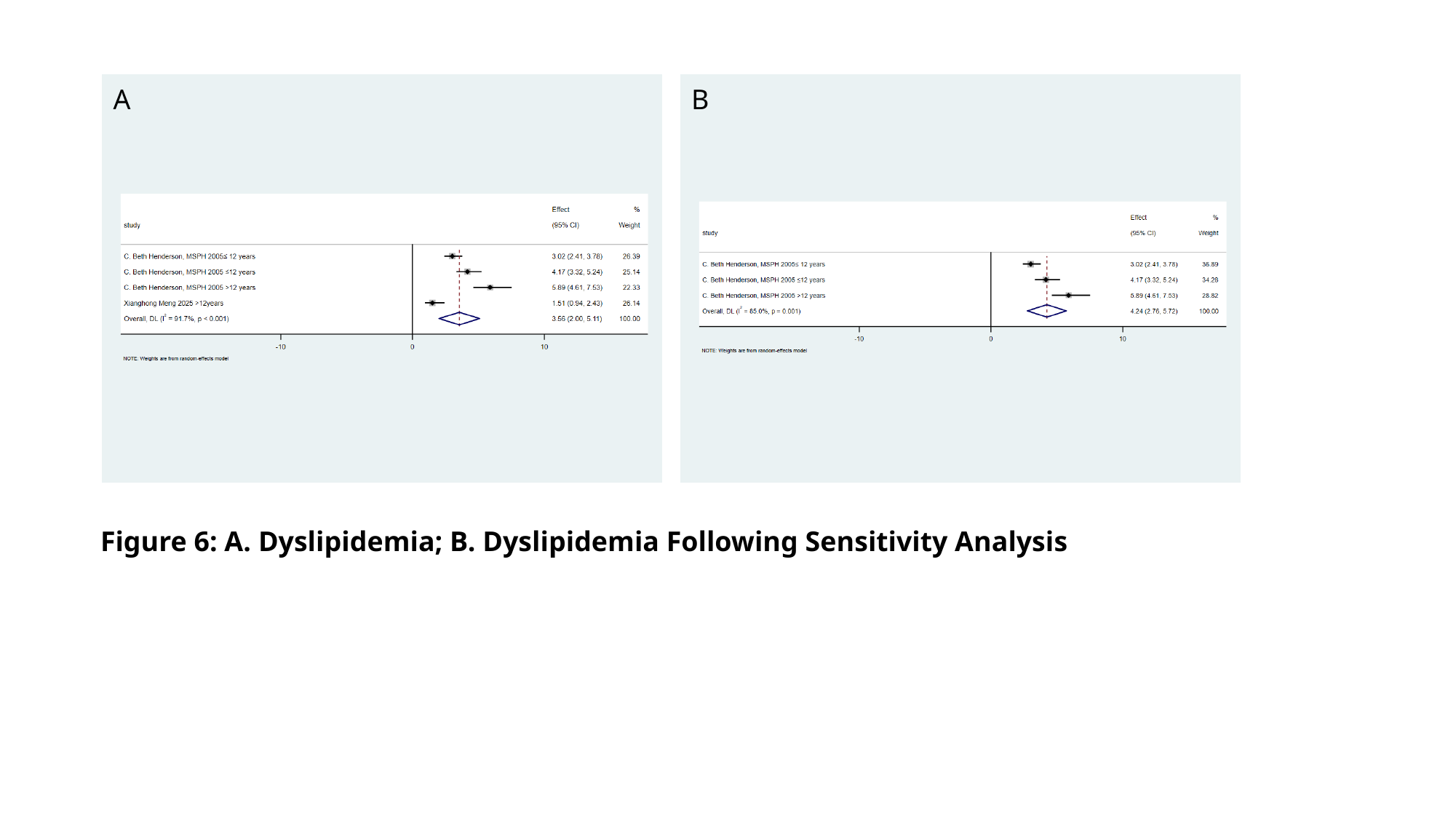

A
B
Figure 6: A. Dyslipidemia; B. Dyslipidemia Following Sensitivity Analysis

## Slide 10
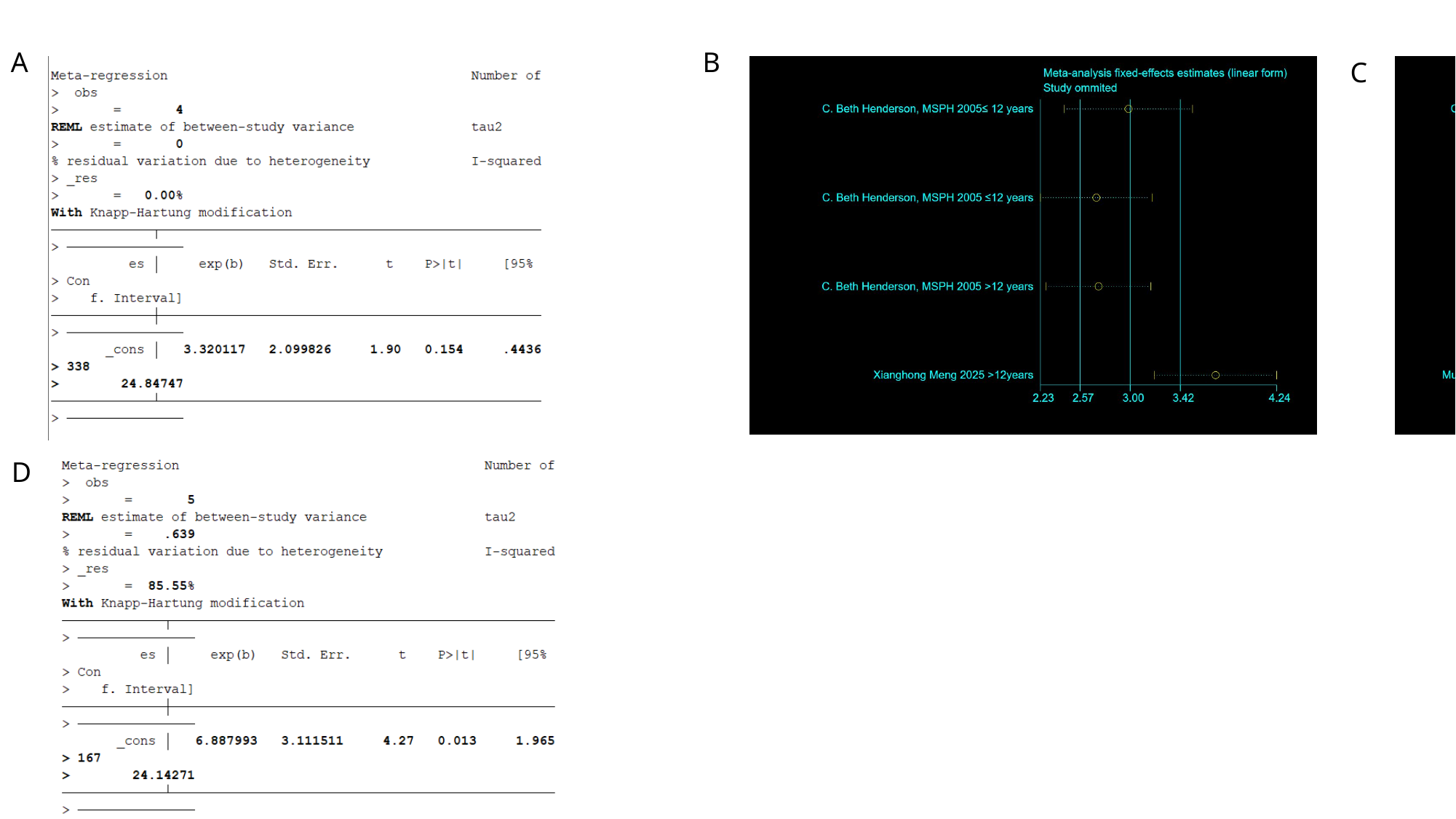

A
B
C
D
Supplementary Figure 4: A. Dyslipidemia Subgroup Analysis; B. Dyslipidemia Sensitivity Analysis; C. Body Weight Reduction; D. Constipation Subgroup Analysis

## Slide 11
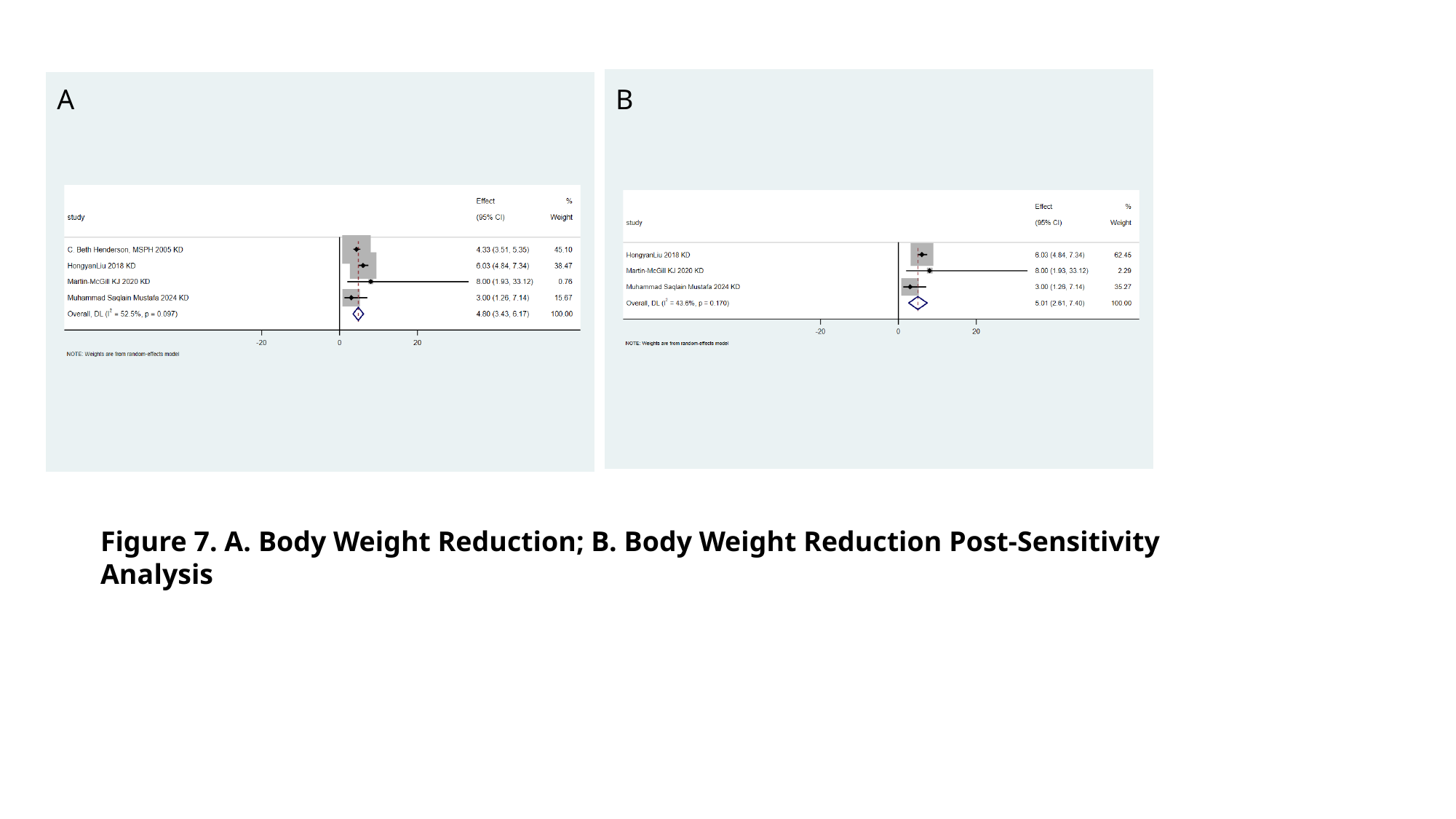

A
B
Figure 7. A. Body Weight Reduction; B. Body Weight Reduction Post-Sensitivity Analysis

## Slide 12
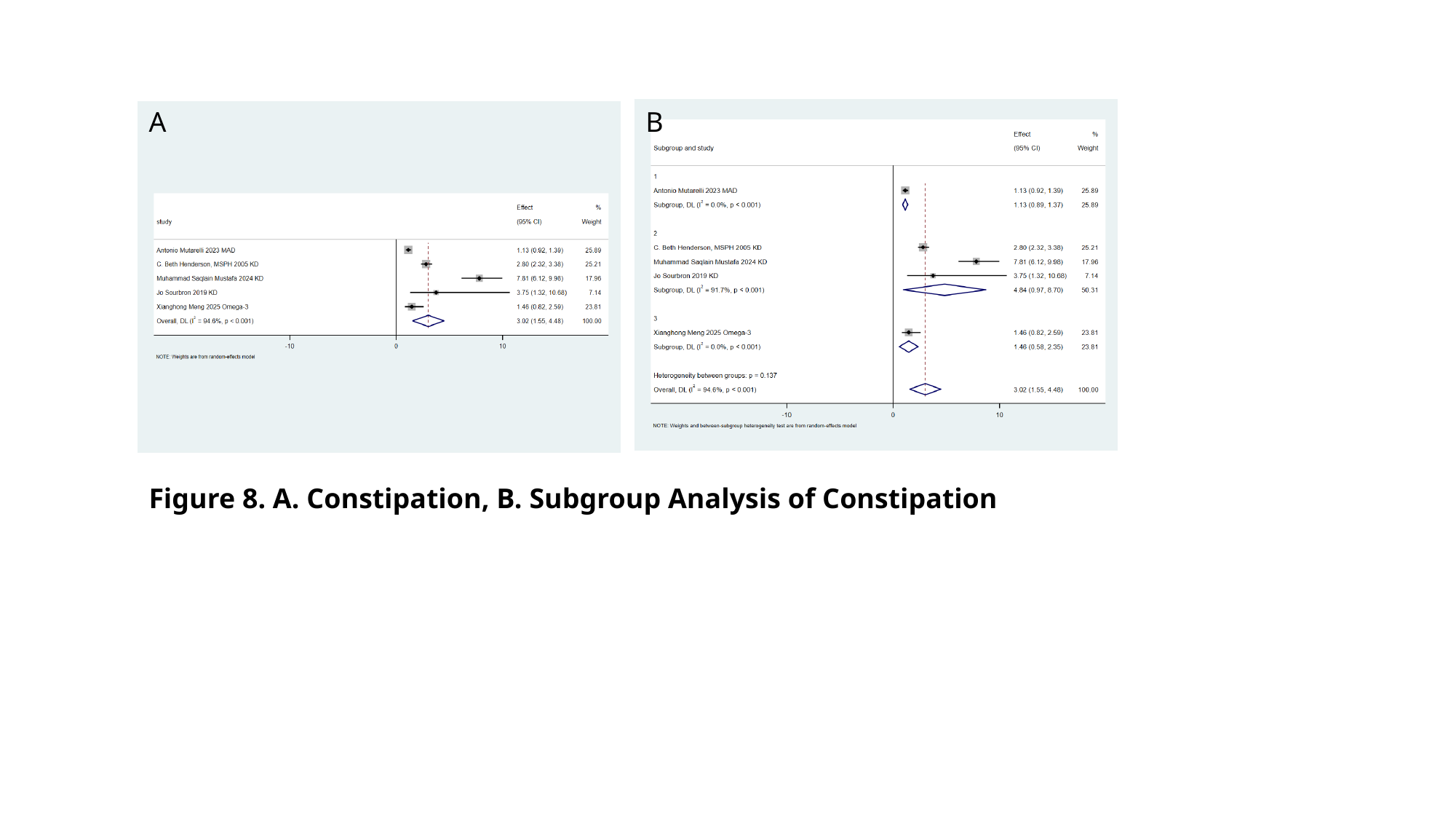

A
B
Figure 8. A. Constipation, B. Subgroup Analysis of Constipation

## Slide 13
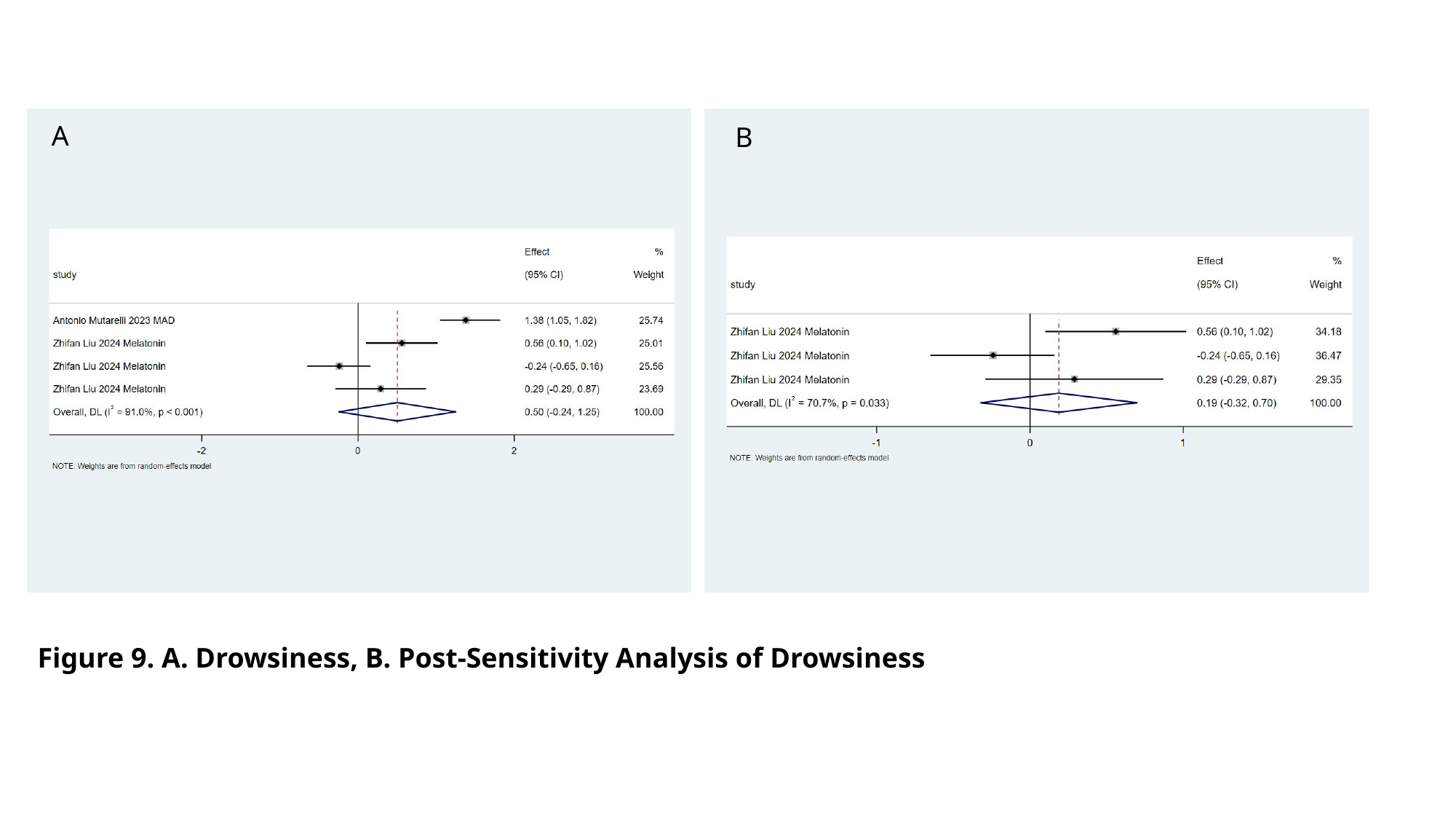

A
B
Figure 9. A. Drowsiness, B. Post-Sensitivity Analysis of Drowsiness

## Slide 14
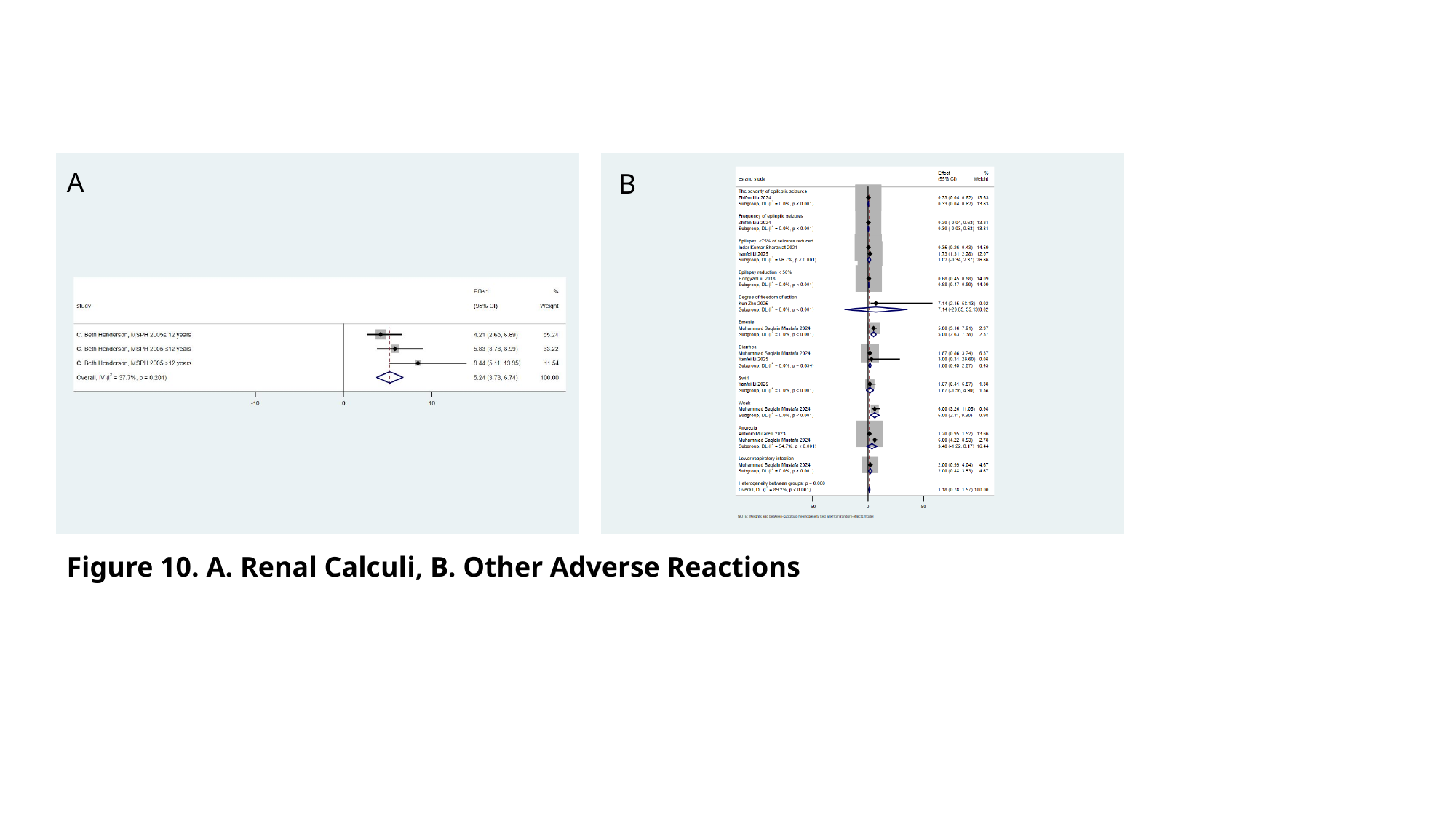

A
B
Figure 10. A. Renal Calculi, B. Other Adverse Reactions
