# Supplementary material for: Efficacy and safety of antioxidants and dietary therapies for epilepsy: an umbrella meta-analysis
Source: Front Nutr. 2026 Jan 12;12:1723370. doi: 10.3389/fnut.2025.1723370 (PMC12832287; doi:10.3389/fnut.2025.1723370)
Supplement: Supplementary file 1 [file Data_Sheet_1.ZIP › Supplementary files/Table S1.docx]

**Table S1 Characteristics of the meta-analysis investigating the impact of inositol supplementation on gestational diabetes**

| **Ref.** | **Participants**  **and case** | **Study**  **number** | **Therapy** | **Intervention time** | **Outcome** | **Quality** |
| --- | --- | --- | --- | --- | --- | --- |
| Mutarelli,A et al**[**20**]** | Adult and pediatric patients with drug-resistant epilepsy | 6 RCTs (575 patients) | Modified Atkins Diet (MAD) | Average 12 weeks (range 4–24 weeks) | ≥50% seizure reduction; seizure freedom; ≥90% seizure reduction; adverse events (constipation, lethargy, anorexia) | Moderate to High (all RCTs; open-label; some with risk of bias concerns) |
| Henderson, C. B et al**[**21**]** | Pediatric patients with intractable epilepsy | 19 (1084 patients) | Ketogenic Diet (Classic or MCT) | Variable (6-48 months) | >50% seizure reduction; complete seizure control; >90% seizure reduction; reasons for dropout | Yes (systematic review, meta-analysis); No (all studies Class III/IV, no blinding or RCTs); Low/Moderate |
| Pizzo, F et al**[**22**]** | Pediatric patients with drug-resistant epilepsy | 8 RCTs (453 patients) | Ketogenic Diet (KD) / Modified Atkins Diet (MAD) | ≥3 months (range 1–24 months) | ≥50% seizure reduction; seizure freedom; cognitive/behavioral improvements; adverse events | Moderate to High (all RCTs; QUADAS-2 low risk; AMSTAR high-moderate quality) |
| HongyanLiu et al**[**23**]** | Adult patients with intractable epilepsy | 16 observational studies (338 patients) | Ketogenic Diet (KD) and variants (CKD, MAD, LGID, LFOD) | Variable (1–40 months) | Seizure freedom (13%); ≥50% seizure reduction (53%); <50% seizure reduction (27%); common adverse events (e.g., weight loss, hyperlipidemia) | Moderate (Observational studies; NOS quality assessment; some heterogeneity) |
| Sharawat, I. K et al**[**24**]** | Children with Lennox-Gastaut syndrome (LGS) | 30 studies (23 CC, 7 KD; 621 patients) | Corpus Callosotomy (CC) and Ketogenic Diet (KD) | Variable (CC long-term; KD ~6 months) | Seizure freedom (CC 40.8%, KD 11.7%); ≥75% reduction (CC 60.0%, KD 34.8%); ≥50% reduction (CC 86.0%, KD 72.0%); AE rate (CC 24%, KD 19.4%) | Moderate (Observational studies; ROBINS-I moderate risk; no direct comparison) |
| Zhang, J et al**[**25**]** | Patients with CDKL5-related epilepsy | 12 retrospective studies (193 patients) | Ketogenic Diet (KD) | Variable (follow-up 3–24 months) | Definite responder rate (≥50% seizure reduction): 18.0%; Clinical responder rate: 50.5%; High GI adverse events (e.g., constipation, vomiting) | Yes (case selection, outcome extraction completeness); no (random allocation, blinding, sample size uniformity); low (all retrospective studies, Newcastle-Ottawa Scale score 3-5) |
| Jo Sourbron et al**[**23**]** | Children and adolescents with refractory epilepsy | 5 RCTs (7 publications, 472 patients) | Ketogenic Diet (KD) and Modified Atkins Diet (MAD) | 3–16 months | ≥50% seizure reduction: 35–56.1% (intervention) vs. 6–18.2% (control); RR = 5.1 (95% CI 3.18–8.21); High GI adverse events (e.g., constipation, vomiting); No severe adverse effects reported | Yes (selection bias control, outcome evaluation); no (blinding, multi-center design); moderate (Cochrane risk of bias tool, most single-center, partial loss to follow-up) |
| Kun Zhu et al**[**27**]** | Patients with drug-resistant epilepsy (DRE) | 17 RCTs (1468 patients) | KD, MAD, LGIT | 4 weeks to 6 months | >50% seizure reduction; >90% seizure reduction; seizure freedom; Compliance; GI adverse events (e.g., constipation, | Yes (allocation concealment, outcome extraction completeness); no (blinding for dietary intervention); high to moderate (ROB2 tool: 47% low risk, 35% some concerns, 18% high risk) |
| Ranganathan, L. N et al**[**28**]** | Patients with epilepsy, 808 cases (706 cases analyzed, including RCTs and quasi-RCTs) | 15 (including RCTs and quasi-RCTs) | 1-Folic acid; 2-Thiamine; 3-Vitamin D; 4-Vitamin E (all as add-on to antiepileptic drugs, AEDs) | Follow-up for 3-12 months | Primary outcome: Vitamin D improved bone mineral content (OR 3.6, 95%CI 2.48-4.72, p<0.00001); vitamin E reduced seizure frequency (p=0.00005); folic acid had no significant effect on seizure control; Secondary outcome: Thiamine improved neuropsychological functions; no | No (randomization concealment, blinding); yes (basic outcome recording); low (small sample size, poor methodological reporting, high risk of bias) |
| Manral, M et al**[**29**]** | Adolescents/adults with DRE, 142 cases (3 RCTs) | 3 RCTs | MAD (carb 15-20g/d) | 2-6 months | ≥50% seizure reduction: pooled prop 0.23, RR 6.47 (p<0.05) | Yes (Jadad score 3, low risk of bias in randomization and patient accountability); no (blinding); high quality (all RCTs with low bias risk) |
| Martin-McGill, K. J et al**[**30**]** | DRE (children 711, adults 221; total 932) | 13 RCTs | KD (classic 4:1/MCT-KD), MAD (10-20g carb/d) | 3-16 months | Children: KD vs usual care - seizure freedom RR3.16, ≥50% RR5.80; adults: no seizure freedom | Yes (random sequence generation, allocation concealment); no (blinding of participants/outcome assessors); low to very low certainty (GRADE: high risk of bias, small sample size) |
| Abbasi, M. M et al**[**31**]** | All-age epilepsy (38 SR/meta; 29-2258 cases) | 38 SR/meta | CKD, MAD, MCT-KD, LGID | 1-24 months | CKD: 3m ≥50% red 53%; MAD: 3m 52%; LGID less effective | Yes (adhered to PRISMA-ScR, registered in PROSPERO); no (uniform quality assessment of included studies); moderate (heterogeneity in included study quality) |
| Mustafa, M. S et al**[**32**]** | Children with DRE (1-18y), 788 cases | 11 RCTs | KD, MAD, LGID | 1-12 months (mean 5.4m) | ≥50% OR6.68, ≥90% OR4.37, seizure freedom OR4.13 | Yes (random sequence generation, allocation concealment, incomplete outcome data control); no (blinding); moderate (Cochrane risk of bias tool, low risk of selection bias) |
| Devi, N et al**[**33**]** | Children with DRE, 907 cases (676 dietary intervention, 257 usual care) | 12 (RCTs) | 1-KD; 2-MAD; 3-LGIT; 4-usual care | Short-term (≤3m), intermediate (4-6m), long-term (12m) | ≥50%/≥90% seizure reduction, AE-related withdrawal | Yes (random, allocation concealment); No (blinding); Low-moderate |
| Liu, X. Y et al**[**34**]** | Children with intractable epilepsy, 1062 cases | 18 | KD (classic, MAD, MCT, etc.) | 3m, 6m | ≥50%/≥90% seizure reduction, seizure freedom | Yes (selection, follow-up); No (blinding); Moderate |
| Meng, X et al**[**35**]** | Patients with DRE (adults+children), 531 cases (9 RCTs) | 9 (RCTs) | 1-Omega-3 (fish oil); 2-placebo | 1-12m | Monthly seizure frequency | Yes (random, allocation); No (partial blinding); Moderate-low |
| Yanfei Li et al**[**36**]** | Patients with epilepsy (mostly children/drug-resistant), 824 cases | 11 (RCTs) | Vitamin E (400IU-600IU/d) + AEDs | 4-24 weeks | ≥75% seizure reduction (RR=1.73), children ≥50% reduction (RR=1.69); T-Aoc↑, MDA↓; no AE difference | Yes (random, outcome completeness); No (blinding); Moderate-low |
| Liu, Z et al**[**37**]** | Patients with epilepsy (children/adults), 301 cases | 10 (RCTs) | Melatonin (3-12mg/d) + AEDs | 5-18 weeks | Sleep latency↓ (RR=0.56), seizure severity↓ (RR=0.33); few AEs | Yes (double-blind, random); No (partial allocation concealment); Moderate |
